# Identification of mouse and human embryonic pancreatic cells with adult Procr^+^ progenitor transcriptomic and epigenomic characteristics

**DOI:** 10.3389/fendo.2025.1543960

**Published:** 2025-02-13

**Authors:** Ana C. Heidenreich, Lucas Bacigalupo, Martina Rossotti, Santiago A. Rodríguez-Seguí

**Affiliations:** ^1^ Instituto de Fisiología, Biología Molecular y Neurociencias (IFIBYNE), CONICET-Universidad de Buenos Aires, Buenos Aires, Argentina; ^2^ Departamento de Fisiología, Biología Molecular y Celular, Facultad de Ciencias Exactas y Naturales, Universidad de Buenos Aires, Buenos Aires, Argentina

**Keywords:** pancreas, beta cell, PROCR, progenitor, RNA-seq, single-cell, ATAC-seq, embryonic

## Abstract

**Background:**

The quest to find a progenitor cell in the adult pancreas has driven research in the field for decades. Many potential progenitor cell sources have been reported, but so far this is a matter of debate mainly due to reproducibility issues. The existence of adult Procr^+^ progenitor cells in mice islets has been recently reported. These were shown to comprise ~1% of islet cells, lack expression of Neurog3 and endocrine hormones, and to be capable of differentiating into all endocrine cell types. However, these findings had limited impact, as further evidence supporting the existence and function of Procr^+^ progenitors has not emerged.

**Methods and findings:**

We report here an unbiased comparison across mouse and human pancreatic samples, including adult islets and embryonic tissue, to track the existence of Procr^+^ progenitors originally described based on their global gene expression signature. We could not find Procr^+^ progenitors on other mouse or human adult pancreatic islet samples. Unexpectedly, our results revealed a transcriptionally close mesothelial cell population in the mouse and human embryonic pancreas. These *Procr-like* mesothelial cells of the embryonic pancreas share the salient transcriptional and epigenomic features of previously reported Procr^+^ progenitors found in adult pancreatic islets. Notably, we report here that *Procr-like* transcriptional signature is gradually established in mesothelial cells during mouse pancreas development from E12.5 to E17.5, which has its largest amount. Further supporting a developmentally relevant role in the human pancreas, we additionally report that a transcriptionally similar population is spontaneously differentiated from human pluripotent stem cells cultured *in vitro* along the pancreatic lineage.

**Conclusions:**

Our results show that, although the previously reported Procr^+^ progenitor cell population could not be found in other adult pancreatic islet samples, a mesothelial cell population with a closely related transcriptional signature is present in both the mouse and human embryonic pancreas. Several lines of evidence presented in this work support a developmentally relevant function for these *Procr-like* mesothelial cells.

## 1 Introduction

A deficit in the quantity of functional β-cells results in insulin deficiency, elevated blood glucose levels, and the onset of the diabetic condition (1). One of the current research focuses is aimed at artificially generating insulin-producing cells to suppress the need for insulin administration in such cases. Although these cells can be successfully derived from human pluripotent stem cells, the efficiency of the protocols still requires optimization (2). An alternative, more ambitious approach, consists in eliciting β-cell regeneration from other cell types of the body (3). The existence of facultative pancreatic progenitors in the adult pancreas has been a matter of debate for decades (4, 5). The cell of origin in adult tissue has been proposed long ago to reside within the ductal tree (6) and, to date, this remains an intensive field of research, as highlighted by recent reports (7–17). However, the only widely accepted non-β cell source for new insulin producing cells is the interconversion of α- δ- and γ-cells under very specific conditions (18–21). Recently, a new adult progenitor population resident in the mouse pancreatic islets has been described, with the potential to replenish all the endocrine cells (22). This population, originally identified by its single-cell RNA-seq (scRNA-seq) global transcriptional profile, is characterized by the co-expression of epithelial (*Epcam*, *Cldn10*) and mesenchymal (*Vim*, *Col3a1*) markers, along with the novel progenitor markers *Procr*, *Rspo1* and *Upk3b*, among others. Procr^+^ progenitor cells were reported to comprise about 1% of adult pancreatic islet cells, lack expression of *Neurog3* or endocrine hormones, and give rise in organ homeostasis to all endocrine cells of the islets. When purified and co-cultured *in vitro* with endothelial cells, Procr^+^ progenitors differentiate into all endocrine cell types and create functional micro-islets that are capable of reversing diabetes in streptozotocin-induced mice (22). However, this groundbreaking report did not have the expected impact in the field, as there were no follow-up reports from independent research groups recapitulating these findings.

Notably, the Procr^+^ progenitor expression profile includes *Rspo1*, *Dcn*, *Upk3b*, and *Procr*, and displays epithelial-to-mesenchymal transition (EMT) features characterized by combined expression of epithelial and mesenchymal markers. It was suggested that these progenitors partially share their transcriptional profile with a subpopulation of endocrine progenitor (EP) cells present in the E14.5 mouse embryonic pancreas, although the major difference is that while the embryonic progenitors express *Neurog3*, their adult counterparts lack expression of this marker (22). Despite the expression of genes associated with EMT is a landmark of the well-established *Neurog3* ^+^ embryonic EP, it should be noted that the embryonic pancreas harbors progenitor cells with diverse transcriptional signatures, many of which are being increasingly characterized through advancements in single-cell RNA sequencing (scRNA-seq) technologies (23–26). Intriguingly, key reported adult Procr^+^ pancreatic progenitor markers (including *Upk3b*, *Rspo1* and *Igfbp5*) were identified in a previous study as characteristic of a pancreatic mesothelial cell population co-purified in scRNA-seq transcriptomes profiled from the mouse embryonic pancreas (23). Procr^+^ progenitors were originally identified from scRNA-seq data profiling both islet-enriched endocrine and non-endocrine cells mixed at a 1:1 ratio (22). Notably, rare progenitor cells of ductal or acinar origin with the potential to give rise to insulin-producing cells have been previously reported (4). Thus, it remains plausible that Procr^+^ progenitors are part of either the ductal or acinar cell compartments.

Taken together, these observations provide a foundation for raising concerns about the actual origin and role of these progenitors. Notably, adult Procr^+^ progenitors have been reported to co-express epithelial markers alongside well-known mesenchymal markers, such as *Col3a1*. While EMT is a prominent feature of EP during pancreas development, cells expressing mesenchymal markers are routinely excluded from integrative analyses tracking developmental trajectories of endocrinogenesis. Consequently, the potential role of rare progenitors with such characteristics remains largely unexplored.

In particular, it is currently unclear whether a *Neurog3*⁻ cell population equivalent to the adult Procr^+^ progenitors exists in the mouse embryonic pancreas, whether such a population is preserved as the pancreas develops, and, more intriguingly, whether these progenitors can also be identified in the developing human pancreas.

In this work, we present the results of an in-depth analysis of scRNA-seq and single-cell ATAC-seq (scATAC-seq) datasets aimed at tracking Procr^+^ progenitor cells in the mouse and human adult islets and in the embryonic pancreas. Our findings reveal that, while these progenitors could not be found in other mouse or human pancreatic islet preparations, in the embryonic pancreas they share an extensive transcriptional profile with a subset of mesothelial cells. Here, we report that these *Procr-like* mesothelial cells can be identified in the mouse and human embryonic pancreas, they are *Neurog3*⁻ and display a transcriptional profile resembling that of the recently identified adult islet-resident Procr^+^ progenitors.

By carefully dissecting the shared gene expression profiles of *Procr-like* mesothelial cells with the previously reported adult Procr^+^ progenitors, and with intermediate epithelial pancreatic progenitor stages connected to the endocrinogenesis process, we provide here a comprehensive annotation of genes potentially relating *Procr-like* mesothelial cells with pancreatic epithelial progenitors during physiological pancreas development in both mice and humans. Notably, we detected these cell clusters in complementary analyses of scRNA-seq datasets obtained from both mouse and human embryonic pancreas samples at several different developmental stages, obtained from separate studies, as well as scATAC-seq data from the mouse embryonic pancreas. Our results suggest a potential developmental lineage relationship: pancreatic bipotent progenitor (BP) → duct→ *Procr-like*/mesothelial, with the latter being overrepresented in late-stage (E17.5) mouse embryonic samples.

## 2 Materials and methods

### 2.1 Single-cell RNA-seq

All analyses were performed using R (v4.1.1), with Conos (27) or Seurat (28) primarily employed for preprocessing, integration, and clustering. A summary of the methods is provided below, with further details available in the **Supplementary Methods**.

#### 2.1.1 Mouse pancreatic islet data integration

Data from six different studies [Gene Expression Omnibus (GEO): GSM2230761 (29); Sequence Read Archive (SRA): SRR10096826 (30), SRR14119316 (31), SRR8754575 (32), SRR11866759 (33); National Omics Data Encyclopedia (NODE): OEP000249, OEP000250 (22)] was dowloaded, re-processed and integrated using Conos and Seurat frameworks. Conos integration employed a graph-based approach, using PCA for dimensionality reduction and angular metrics to construct a shared nearest-neighbor graph. The Leiden algorithm revealed consistent clustering patterns that preserved cell identities and inter-dataset relationships, ensuring robust biological comparisons across datasets while minimizing batch effects. Graph embedding was conducted using the ‘largeVis’ method, resulting in an integrated dataset of 41,049 cells and 32,541 features. Seurat’s canonical correlation analysis (CCA) identified shared anchors across datasets, followed by data integration with IntegrateData(). The integrated dataset underwent scaling, PCA, clustering at a resolution of 1, and UMAP visualization, which preserved biological variability while providing a cohesive view of the datasets.

#### 2.1.2 Human pancreatic islet data integration

Processed datasets from Grün et al. (34), Muraro et al. (35), and Segerstolpe et al. (36) were integrated via Seurat, correcting batch effects with FindIntegrationAnchors(). The final dataset comprised 7,291 cells and 21,329 features.

#### 2.1.3 Human and mouse embryonic pancreas cell data integration

Single-cell RNA-seq data from human embryonic pancreas (GSA: HRA002757, PCW 4-11) and mouse embryonic pancreas (GEO: GSM3140915-18 and GSM3938451, E12.5-E17.5) were integrated to study pancreatic development. Human data, processed with SCTransform and clustered via PCA and Louvain algorithms, retained 28,368 and 44,734 singlets for PCW 4-6 and PCW 7-11, respectively, after doublet removal. Mouse data were normalized, variable genes identified, and gene names converted to human nomenclature for integration. Conos integration used PCA components and over-dispersed genes to construct a shared nearest-neighbor graph. Leiden clustering identified annotated clusters, with additional subclustering for endocrine hormone-expressing cells. Slingshot trajectory analysis revealed differentiation paths from Mesothelial^TBX3+ early^ cells to β-cells, highlighting lineage relationships and developmental dynamics. Visualization with largeVis ensured biological coherence across datasets.

#### 2.1.4 Mouse embryonic pancreas integration

Developmental-stage datasets were preprocessed with Seurat, filtering for quality and regressing cell cycle effects. Datasets were integrated and analyzed using Conos to reveal key progenitor and epithelial populations.

#### 2.1.5 Trajectory analysis

Lineage relationships were inferred using Slingshot (37), identifying potential differentiation dynamics from *Procr-like* mesothelial cells to mature β-cells.

#### 2.1.6 Organoid scRNA-seq analysis

Pre-clustered organoid datasets were analyzed without additional processing, focusing on Procr^+^ progenitor cells and stages of development.

#### 2.1.7 Human *in vitro* pancreatic progenitor data analysis

The human Day 13 pancreatic progenitor single-cell RNA-seq data (GEO: GSM5127847) was processed with Cell Ranger (v2.01) aligned to the GRCh38 genome. Low-quality cells (high mitochondrial content, low gene count) were filtered, and clusters identified using Seurat’s FindNeighbors and FindClusters. Differential expression was analyzed with Wilcoxon tests. Doublets were removed using scDblFinder. The *in vitro* Procr-like cells were integrated and analyzed with human embryonic pancreas mesothelial subsets using the Conos package.

### 2.2 Single-cell ATAC-seq

For single-cell ATAC-seq analysis of the E17.5 mouse embryonic pancreas, data from GEO GSM7244762/63 was processed using Seurat and Signac (38). Quality control metrics, including nucleosome signal and TSS enrichment, were applied to filter low-quality cells. Peak calling was performed independently for each lane and merged using Seurat’s functions. The data were normalized using TF-IDF, followed by dimensionality reduction (SVD) and clustering via the Louvain algorithm. Doublets were excluded using scDblFinder, and peak annotations were assigned using the EnsDb.Mmusculus.v79 database. Integrated data from scRNA-seq and scATAC-seq were visualized with UMAP, with predictions of cell types transferred from scRNA-seq based on gene activity profiles. Motif discovery was conducted using HOMER (39) on key chromatin regions. Additional details on scATAC-seq data processing and analysis can be found in the **Supplementary Methods**.

### 2.3 Statistical analysis

Statistical significance was assessed using Wilcoxon rank-sum test with *P* < 0.05 considered significant. All statistical analysis and visualization were done with R and Bioconductor package.

## 3 Results

### 3.1 Procr^+^ progenitors are not found in other adult pancreatic islet samples

To track the presence of Procr^+^ progenitors originally identified in adult mouse pancreatic islets (22) (hereafter referred to as Procr^+^ progenitors), we integrated the original dataset generated by Wang et al. with five previously reported adult mouse pancreatic islet scRNA-seq samples (29–33). To unequivocally track the same cells, we retained the cell labels originally reported (data kindly provided by the authors) (22). Since identifying equivalent cell clusters across different datasets can be biased by the data integration strategy, we evaluated two broadly validated scRNA-seq integration approaches in parallel: the Conos and Seurat pipelines (27, 28).

**Figure 1:**
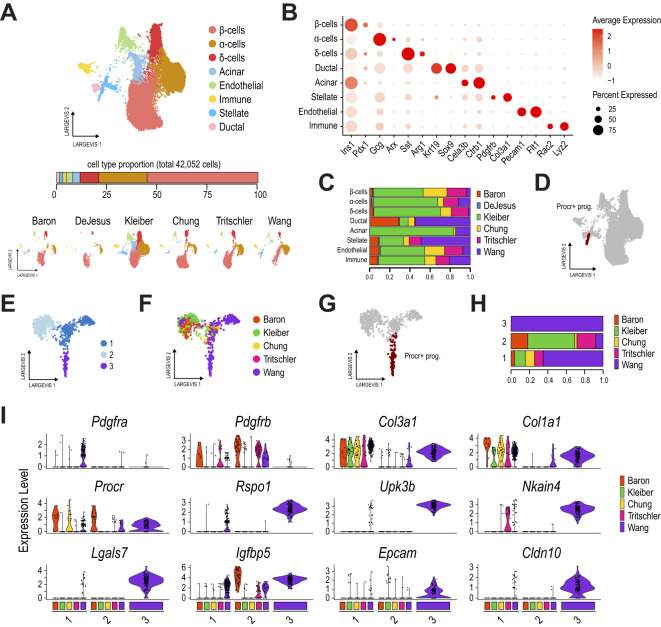
Procr^+^ progenitors are not found in other adult pancreatic islet samples. **(A)** Dimension plots of single-cell transcriptomes profiled from six different mouse adult pancreatic islet datasets, integrated with Conos. The bar below the main plot displays the proportion of each cell type relative to the total number of cells analyzed. Dimension plots in the bottom show cell distribution according to the sample of origin. **(B)** Dot plot showing the expression of key markers for endocrine, ductal, acinar, stellate, immune, and endothelial cell types, used to define clusters. **(C)** Proportion of cells from each dataset contributing to the cell clusters identified in **(A)**. **(D)** Dimension plot highlighting the Procr^+^ progenitor cells originally reported. **(E)** Dimension plot of pancreatic stellate cells reclustered from **(A)** and reanalyzed with Conos. **(F, G)** Dimension plots showing cells colored by the sample of origin **(F)** and highlighting the Procr^+^ progenitor cells originally reported **(G)**. The DeJesus dataset was excluded from this analysis as it contributed an extremely small number of cells. **(H)** Proportion of cells from each dataset contributing to each stellate cell subcluster. **(I)** Violin plots showing the expression of key activated (*Pdgfra*, *Col3a1*, *Col1a1*) and quiescent (*Pdgfrb*) stellate markers, along with Procr^+^ progenitor cell markers (*Procr*, *Rspo1*, *Upk3b*, among others), for each stellate cell subcluster identified in **(E)**, split by the sample of origin. Cluster 3 contains only cells from the Wang et al. dataset.

In total, we integrated 41,049 cells using Conos, and the results revealed 8 cell clusters matching most of the expected pancreatic cells in this tissue (**Figures 1A, B**, **Supplementary Table 1**), i.e. α- (*Gcg* ^+^), β- (*Ins1* ^+^) and δ-cells (*Sst* ^+^), as well as ductal (*Sox9* ^+^, *Krt19* ^+^), acinar (*Cela3b* ^+^), stellate (*Pdgfrb* ^+^, *Col3a1* ^+^), endothelial (*Pecam1* ^+^) and immune cells (*Rac2* ^+^). Of note, we could track cells from all samples in most clusters (**Figure 1C**). The Procr^+^ progenitors (as originally labeled, **Figure 1D**) were clustered with pancreatic stellate cells in this global analysis, likely due to the extensive overlap in the expression of mesenchymal/stellate markers.

An in-depth analysis of the stellate cluster identified three subclusters (**Figure 1E**). While clusters 1 and 2 contained cells from all samples, cluster 3 comprised exclusively Procr^+^ progenitor cells from the Wang et al. dataset (**Figures 1F–H**). A detailed analysis of stellate and Procr progenitor markers, split by sample of origin for each cluster, distinguished Procr^+^ progenitors from quiescent (*Pdgfrb*+/*Col1a1*-) and activated (*Pdgfra*+/*Col1a1*+) stellate cells (**Figure 1I**). Interestingly, except for *Ifgbp5* and *Procr* itself, cells in cluster 3 expressed Procr^+^ progenitor markers at high levels and these were largely undetected in cells of clusters 1 and 2, consisting of quiescent and activated stellate cells (**Figure 1I**). The scarce number of cells expressing Procr markers at intermediate levels in the stellate subclusters does not entirely exclude the possibility that these could represent Procr-like cells present at extremely low frequencies in other samples. Nevertheless, the unbiased clustering analysis indicates that these cells exhibit a global transcriptional profile more similar to stellate cells than Procr^+^ progenitors. Importantly, similar results were obtained using the Seurat integration approach (**Supplementary Figures 1A–J**). Taken together, our results suggest that, despite being transcriptionally similar to pancreatic stellate cells, Procr^+^ progenitors are not detected in other adult mouse pancreatic islet samples.

The integration of mouse (22) and human pancreatic islet datasets (34–36) allowed the identification of equivalent cell clusters as described above, matching most of the expected pancreatic cells in this tissue (**Supplementary Figures 1K–M**, **Supplementary Table 2**). An in-depth analysis of the stellate cluster in this cross-species integrated dataset also yielded similar results, mapping Procr^+^ progenitors to the stellate cluster (**Supplementary Figure 1N**). Consistent with our earlier analysis, a detailed inspection of this cluster identified three subclusters associated with quiescent and activated stellate cells (clusters 0 and 1, respectively; **Supplementary Figures 1O–T**), and Procr^+^ progenitors (cluster 2). Notably, although cluster 2 contained a small number of cells from the human datasets, expression analysis of stellate and progenitor markers, split by sample of origin, revealed that cells in cluster 2 from human datasets did not express Procr+ markers such as *UPK3B*, *RSPO1* and *LGALS7* (**Supplementary Figure 1T**).

### 3.2 Mesothelial cells in the mouse embryonic pancreas exhibit a transcriptional profile closely resembling that of adult Procr^+^ progenitors

We next interrogated whether Procr^+^ progenitors could be detected during mouse pancreas development. For this purpose we integrated the original adult islet dataset generated by Wang et al. with mouse embryonic pancreas scRNA-seq datasets profiled at different developmental stages, from E12.5 to E17.5 (23, 40). As described above, we kept the Procr^+^ progenitor cell labels originally reported. Additionally, to improve the accuracy of our integration analysis, we carefully excluded potential doublets (see *Methods*).

Since several progenitor cell types in the embryonic pancreas lack counterparts in the adult pancreatic islet sample, we employed a recently reported integration strategy (Conos) to accommodate clusters not present in all samples (27). Integration of the mouse adult islet dataset containing Procr^+^ progenitors with E12.5, E13.5, E14.5 and E17.5 datasets included in total 36,739 cells after quality filtering and doublet removal. A cell clustering analysis revealed most of the expected cell types in the developing pancreas, including mesenchymal (*Col3a1* ^+^), endothelial (*Pecam1* ^+^), immune (*Rac2* ^+^), erythrocytes (*Hba-a1* ^+^) and neural-crest-derived/Schwann cells (*Sox10* ^+^), besides the epithelial cells (*Epcam* ^+^) accounting for the different progenitor and differentiated pancreatic cell types (**Supplementary Figures 2A, B**, **Supplementary Table 3**). Notably, Procr^+^ progenitors (as labeled by Wang et al.) were mapped to cluster 2 (**Supplementary Figure 2C**), which also included cells from all other pancreatic embryonic stages (**Supplementary Figure 2D**). Unlike mesenchymal cells, this cluster, along with the transcriptionally similar cluster 10, expressed epithelial markers (e.g., *Krt19*, and low levels of *Epcam*), as well as previously reported Procr^+^ progenitor marker genes such as *Procr*, *Rspo1*, *Upk3b* and *Igfbp5* (**Supplementary Table 3**). Unexpectedly, these clustered cells expressing *Upk3b*, *Rspo1* and *Igfbp5* in the embryonic pancreas have been previously classified as mesothelial cells based on their co-expression with other established mesothelial markers such as *Wt1*, *Krt19* and *Msln* (23). However, a detailed characterization of the expression pattern of these genes, associated with adult Procr^+^ progenitors, has not been reported for the developing mouse pancreas. Notably, given that *Krt19* is also a well-known epithelial pancreatic ductal marker (41), and that other classic mesenchymal markers, such as *Vim*, are also expressed in pancreatic epithelial cells undergoing migration during development (42), it remains plausible that low-level expression of *Upk3b*, *Igfbp5*, and other Procr^+^ marker genes is shared among various cell populations within the embryonic pancreas (**Supplementary Figure 2E**). Supporting a potential role for *Upk3b* in epithelial pancreatic progenitors, recent findings indicate that *Upk3bl1*, an important paralog for *Upk3b*, is upregulated in EP at advanced stages of endocrinogenesis (43).

**Figure 2:**
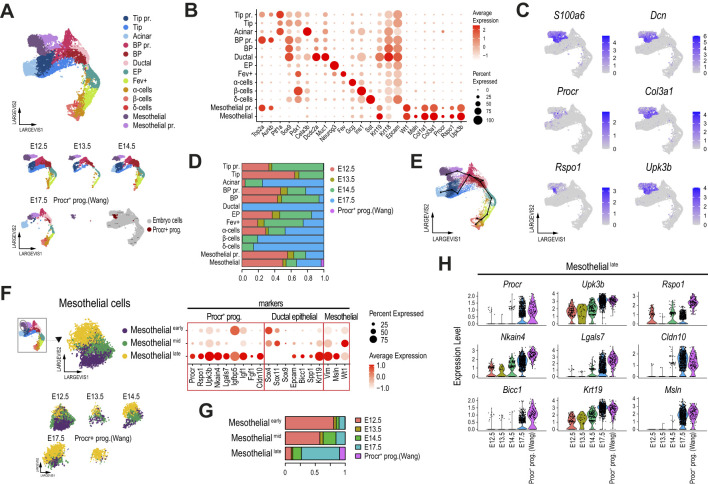
Mesothelial cells in the mouse embryonic pancreas exhibit a transcriptional profile closely resembling that of adult Procr^+^ progenitors. **(A)** Dimension plot of single-cell pancreatic epithelial and mesothelial transcriptomes profiled from the E12.5-E17.5 mouse embryonic pancreas, integrated using Conos with the originally reported Procr^+^ progenitor cells profiled from adult pancreatic islets. Dimension plots in the bottom panel show cell distribution according to sample timepoint. The bottom right dimension plot highlights the location of the originally reported Procr^+^ progenitors. **(B)** Dot plot showing the expression of selected pancreatic and Procr^+^ progenitor markers used to match cell clusters. Cells clustered as in **(A)**. **(C)** Feature plots showing the expression of selected Procr^+^ progenitor cell markers. **(D)** Proportion of cells from each dataset contributing to the cell clusters identified in **(A)**. **(E)** Trajectory analysis ordering of mesothelial and pancreatic epithelial cells clustered as shown in **(A)**. **(F)** Dimension (left panels) and dot (right panel) plots showing re clustering of mesothelial cells from **(A)**. Dimension plots in the bottom panel show cell distribution according to sample timepoint. The dot plot shows the expression of selected Procr^+^ progenitor cell markers, genes with relevant functions in ductal and other pancreatic epithelial cells, and mesenchymal/mesothelial-associated genes, as indicated in the labels. **(G)** Proportion of cells from each dataset contributing to the cell clusters identified in **(F)**. **(H)** Violin plots showing the expression of key Procr^+^ progenitor cell markers in cells of the mesothelial^late^ cluster (as defined in panel **F**), split by developmental timepoint.

To further explore the potential lineage relationship of a subset of these mesothelial cells (with the Procr^+^ progenitor expression pattern) and various pancreatic progenitor cell types, we selectively reanalyzed the pancreatic epithelial cells (red box clusters in **Supplementary Figure 2B**), the mesothelial cells matching the Procr-like expression profile (clusters 2 and 10), and the adult Procr^+^ progenitor cells. This approach allowed us to more precisely identify most of the epithelial pancreatic progenitor and differentiated cell types expected at these developmental stages based on established cell type markers (24, 25, 44–46) (**Figures 2A, B**). These included tip (*Ptf1a* ^+^ *, Sox9* ^+^ *, Pdx1* ^+^) and trunk/bipotent progenitor (BP; *Sox9* ^+^ *, Pdx1* ^+^ *, Dcdc2* ^+^) cells, which were additionally subset upon the co-expression of proliferative genes (*Top2a* ^+^, *Aurkb* ^+^), as well as acinar (*Cela3b^+^*), ductal (*Sox9* ^+^, *Muc1* ^+^), EP (*Neurog3* ^+^), Fev^+^ progenitors (*Fev* ^+^), α-cells (*Gcg* ^+^), β-cells (*Ins1* ^+^) and δ-cells (*Sst* ^+^) (**Figure 2B**, **Supplementary Table 4**). We also identified two additional clusters that co-expressed epithelial (*Krt18* ^+^, *Krt19* ^+^) (41) and mesenchymal (*Col3a1* ^+^) markers, which were additionally subset upon the co-expression of proliferative genes (*Top2a* ^+^, *Aurkb* ^+^). These also co-expressed most of the previously reported Procr^+^ progenitor markers, including *Procr*, *Rspo1* and *Upk3b* (**Figures 2B, C**). Since the cells in these clusters also expressed the mesothelial cell markers *Wt1* and *Msln*, and were previously associated with this cell type, we hereafter refer to these *Procr-like* clusters as “Mesothelial” and “Mesothelial proliferative” (Mesothelial pr.).

Of note, Procr^+^ progenitors from adult pancreatic islets mapped to the Mesothelial cluster, and this cluster also included cells from all embryonic pancreatic stages analyzed (**Figure 2D**). Thus, the previously reported adult Procr^+^ progenitor population (22) presents a global transcriptional profile that matches a subset of cells previously associated with mesothelial cells (23), and this subpopulation can be tracked in samples of the E12.5-E17.5 mouse embryonic pancreas profiled from 2 independent studies (23, 40). Although these clusters did not directly overlap with pancreatic epithelial cells (**Supplementary Figure 2F**), a trajectory analysis associated them with ductal and BP cells (**Figure 2E**), suggesting the provocative hypothesis that they are transcriptionally related to cells of ductal origin. The genes transcriptionally linking mesothelial clusters with ductal cells included previously reported ductal and BP marker genes such as *Anxa2*, *Spp1*, and *Bicc1* (25, 47, 48). Interestingly, this also included genes involved in ductal-mediated regenerative processes such as *Clusterin* (*Clu*) (49). However, the well-known BP/ductal lineage markers *Sox9* and *Hnf1b* were not expressed in mesothelial cells (**Supplementary Figure 2G**). Importantly, most of these genes were preferentially expressed in pancreatic epithelial cells rather than mesenchymal cells (**Supplementary Figure 2H**).

An in-depth analysis of the Mesothelial cluster revealed three interconnected subclusters (**Figure 2F**), which were named Mesothelial^early^, Mesothelial^mid^ and Mesothelial^late^, based on their proportion of cells from the early (E12.5/E13.5), mid (E14.5), and late (E17.5) pancreatic developmental stages (**Figure 2G**). Interestingly, Procr^+^ progenitors from adult islets mapped to the Mesothelial^late^ cluster, which was largely composed of E17.5 cells. Further supporting a maturing role during pancreas development, we found that the expression of key Procr^+^ progenitor markers increased from E12.5 to E17.5 in cells of the Mesothelial^late^ cluster, with the highest expression levels observed at E17.5, approaching those of *bona fide* Procr^+^ progenitors reported by Wang et al. (**Figure 2H**). Interestingly, we found that *Sox4* and *Sox11*, coding for transcription factors with key previously reported roles in BP and EP commitment (46, 50), were expressed at higher levels in Mesothelial^early^ and Mesothelial^mid^ clusters, respectively (**Figure 2F**, dotplot panel). Noteworthy, while *Sox9* could be barely detected in Mesothelial cluster cells, other previously reported BP/ductal markers were upregulated in Mesothelial^late^ cells, following the expression pattern for *Epcam*. These included *Bicc1*, *Spp1* and *Krt19* (24, 41, 48, 51). Finally, we detected upregulation of *Vim* (a mesenchymal marker also upregulated in the BP→EP transition), *Msln*, and *Wt1* (mesothelial markers) (23) in the Mesothelial^mid^ (*Wt1*) and Mesothelial^late^ (*Vim*, *Msln*) stages. While *Wt1* is also expressed in activated pancreatic stellate cells (PaSCs) and may play key roles in pancreatic regeneration (52), its potential role during pancreas development remains to be explored.

Although we accounted for doublets in cells from the embryonic pancreas (53), cells in the Mesothelial clusters exhibited a combination of markers that play key roles in pancreatic epithelial progenitors (*Sox4*, *Bicc1*, *Spp1*) and mesothelial markers (*Msln*, *Wt1*), along with high expression levels of previously reported markers of adult Procr^+^ progenitors (*Procr*, *Upk3b*, and *Igfbp5*, and others). While some of these factors (e.g., *Sox4*, *Krt19*) are expressed and play relevant roles in both pancreatic epithelial and mesenchymal/mesothelial cells (41, 46, 50, 52, 54), others have been primarily reported in the context of pancreatic epithelial BP/ductal lineage commitment (e.g., *Bicc1*, *Spp1*). This led us to evaluate the expression of these markers in the original adult pancreatic islet dataset (**Supplementary Figure 2I**) (22). Interestingly, we found that the originally reported Procr^+^ progenitors were the only identified cell clusters that expressed *Msln* and *Upk3b*, while the expression of other markers in Procr^+^ progenitors was selectively shared with Stellate and Endothelial (*Procr*), Ductal (*Krt19*, *Sox4*), Stromal (*Wt1*), or Ductal and Stromal cells (*Bicc1*, *Vim*) (**Supplementary Figure 2J**). *Vim* was also expressed in Stellate, Endothelial and Immune cells. Taken together, these results suggest that a developmental lineage potentially giving rise to adult Procr^+^ progenitors is established early in mouse pancreas development.

### 3.3 The chromatin accessibility landscape of a subset of mesothelial cells supports epithelial pancreatic progenitor competence

Our results suggest that the adult Procr^+^ progenitor lineage is gradually established during mouse pancreas development, with mesothelial cells from the E17.5 mouse embryonic pancreas exhibiting the closest transcriptional profile. However, while these cells are characterized by co-expression of epithelial and mesenchymal/mesothelial markers, the expression levels of other key pancreatic progenitor markers, such as *Pdx1*, *Sox9*, or *Ptf1a*, remain undetectable. Additionally, these cells, which exhibit a transcriptional profile highly similar to that of adult Procr+ progenitors (*Upk3b* ^+^, *Rspo1* ^+^, among other markers), were previously classified as mesothelial cells (23). Thus, the competence of at least a subset of these cells to give rise to epithelial pancreatic progenitors and endocrine cells remains uncertain.

One known limitation of scRNA-seq is the potential misinterpretation of results due to the sequencing of doublets (which we aimed to reduce in our analysis by using a state-of-the-art approach) (53), as well as contamination with ambient RNA (which is most relevant for highly expressed genes). Analysis of chromatin accessibility at the single-cell level (single-cell ATAC-seq) provides a complementary approach that may be more robust to this latter bias. Additionally, it has been reported that epigenetic competence in pancreatic progenitors is acquired prior to changes in their corresponding transcriptional profiles (55). Thus, to investigate whether *Procr-like* mesothelial cells present accessible chromatin at key epithelial pancreatic genes, we analyzed the single-cell ATAC-seq profiles (scATAC-seq) of the E17.5 mouse embryonic pancreas (56).

To increase the accuracy of downstream analyses, we used the scDblFinder package to remove doublets (53). A global cell clustering analysis allowed us to discriminate most of the expected cell types in the developing pancreas at this stage based on the calculated gene activity profiles (i.e., accessibility regions located within the gene body and promoter region for each gene). Although with less accuracy, due to the more ubiquitous pattern of accessibility regions, we were able to track mesenchymal (*Col3a1* ^+^), endothelial (*Pecam1* ^+^), immune (*Rac2* ^+^), and neural-crest-derived/Schwann cells (*Sox10* ^+^), in addition to the epithelial cells (*Epcam* ^+^) accounting for the different progenitor and differentiated pancreatic cell types (**Supplementary Figures 3A, B**). We also identified a cell cluster matching mesothelial cells with a Procr-like progenitor gene activity profile (cluster 2).

**Figure 3:**
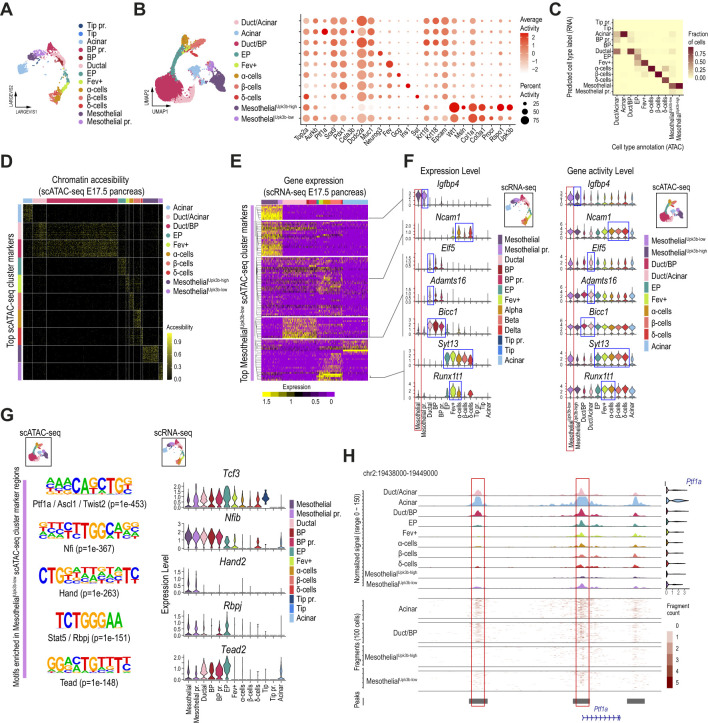
The chromatin accessibility landscape of a subset of mesothelial cells supports epithelial pancreatic progenitor competence. **(A)** Dimension plot of E17.5 single-cell pancreatic epithelial transcriptomes clustered as in **Figure 2A**. **(B)** UMAP (left panel) and dot (right planel) plots of single-cell accessibility profiles obtained for the epithelial and mesothelial cells of the E17.5 mouse embryonic pancreas. The dot plot shows the gene activity scores of selected pancreatic, mesothelial, and Procr^+^ progenitor markers. **(C)** Heatmap showing the fraction of cells from each predicted ATAC cell type (x-axis) corresponding to RNA cell type annotations (y-axis, scRNA-seq analysis presented in A). The color scale represents the fraction of cells for each combination of RNA annotation and predicted ATAC label. **(D)** Heatmap showing chromatin accessibility for the top 50 marker regions across pancreatic epithelial and mesothelial cells of the E17.5 mouse embryonic pancreas, clustered as shown in **(B)**. Each row represents a marker region, and each column represents a cell in the scATAC-seq clusters (Acinar, Duct/Acinar, Duct/BP, EP, Fev+, α-cells, β-cells, δ-cells, Mesothelial^Upk3b-high^, and Mesothelial^Upk3b-low^), with color intensity indicating the level of chromatin accesibility. **(E)** Heatmap showing the scaled expression values for genes associated with the top 1,000 Mesothelial^Upk3b-low^ cluster marker regions recovered from the scATAC-seq analysis. Each row represents a marker region, and each column represents a cell in the scRNA-seq clusters. Color intensity indicates gene expression levels corresponding to pancreatic epithelial and mesothelial cells of the E17.5 mouse embryonic pancreas, clustered as shown in **(A)**. The dendrogram on the y-axis highlights subsets of Mesothelial^Upk3b-low^ cluster marker regions that share expression with specific clusters of the E17.5 scRNA-seq samples. **(F)** Violin plots showing the expression level (left panel) and gene activity score (right panel) of selected genes from each subset of Mesothelial^Upk3b-low^ cluster marker regions identified in **(E)**. **(G)** Highly enriched motifs recovered *de novo* from the Mesothelial^Upk3b-low^ cluster marker genomic accesible regions (left panels). The enrichment p-value along with the top factors matching each motif are displayed below. Violin plots on the right display the expression pattern in cells of the E17.5 embryonic pancreas for selected genes coding for the top transcription factors matching the *de novo* DNA binding motifs shown. **(H)** Chromatin accessibility landscape in the vicinity of *Ptf1a* for E17.5 pancreatic epithelial and mesothelial cells clustered as in **(B)**. The bottom tracks display the fragment count for selected clusters, and the accessibility regions with enrichment over background (peaks). The violin plots on the right display the gene activity score for *Ptf1a* at each cell cluster.

Following the same approach as described for the scRNA-seq analyses, we next selectively reanalyzed the pancreatic epithelial cells (red box clusters in **Supplementary Figure 3B**) and mesothelial cells matching the Procr-like expression profile (cluster 2). We used the scRNA-seq expression profiles of the E17.5 pancreas (**Figure 3A**) from our integrated analysis to label the 10 scATAC-seq clusters identified. This approach allowed us to track most of the expected epithelial pancreatic progenitor and differentiated cell types at this developmental stage based on their average gene activity scores at established cell type markers (**Figure 3B**). Based on their cell type label predicted from the scRNA-seq analysis, we relabeled the scATAC-seq clusters as Duct/Acinar, Acinar, Duct/BP, EP, Fev^+^, α, β, δ, and 2 subsets of *Procr-like* mesothelial cells (**Figures 3B, C**). In contrast with our scRNA-seq classification, the mesothelial clusters identified from the scATAC-seq datasets did not differ in accessibility at cell cycle-associated genes, but rather in their accessibility levels at proper Procr^+^ progenitor marker genes (e.g., *Rspo1*, *Upk3b*). We thus renamed them as Mesothelial^Upk3b-high^ and Mesothelial^Upk3b-low^. Notably, while chromatin accessibility at the *Upk3b* promoter was undetectable in Mesothelial^Upk3b-low^ cells, these displayed increased accessibility at key pancreatic epithelial genes, including *Neurog3*, *Pdx1 and Sox9*, suggesting that this cell subset could be primed for pancreatic epithelial progenitor competence (**Figure 3B**, **Supplementary Figure 3C**).

Subclustering restricted to the Mesothelial^Upk3b-low^ cells identified several subsets, all of which exhibited gene activity at pancreatic epithelial genes (e.g., *Ptf1a*, *Sox9*, *Pdx1*, *Neurod1*) and Procr progenitor markers (e.g., *Procr*, *Rspo1*, *Upk3b*), further supporting a gradual transition in the chromatin accessibility profiles in the vicinity of these genes (**Supplementary Figures 3D, E**). Importantly, top cluster marker regions, considered without any proximity restriction to annotated genes, displayed a strikingly cluster-exclusive accessibility pattern, further supporting that these are *bona fide* distinct cell subtypes and arguing against potential contamination with doublets (**Figure 3D**).

We next examined in closer detail the accessibility pattern of Mesothelial^Upk3b-low^ cells. We followed two approaches for downstream analyses. On one hand, we evaluated the expression profile of genes associated (either overlapping or located within 10Kb of the gene body) with the top 1,000 accessibility marker regions in pancreatic progenitor and differentiated cells as clustered from our scRNA-seq analysis (**Figure 3A**). Out of the 783 genes associated with the top 1,000 Mesothelial^Upk3b-low^ cluster marker regions, 583 were detected in cells of the pancreatic epithelial and/or Mesothelial scRNA-seq clusters of the E17.5 pancreas. We focused on the subset of these which presented a strong and consistent expression, thus retaining 102 genes that presented robust associations between the accessibility regions and its expression in any specific cluster. Noteworthy, while almost one third of these were selectively expressed at high levels in Mesothelial and Mesothelial pr. cells, the remaining genes were expressed at low/undetectable levels in these clusters, and presented expression profiles that were enriched either at Ductal/BP, EP/endocrine and/or Tip/Acinar clusters (**Figure 3E**, **Supplementary Table S5**). Thus, while two-thirds of the genes associated with the top cluster marker regions accessible in Mesothelial^Upk3b-low^ cells are expressed in different subsets of pancreatic epithelial progenitors and/or differentiated cells, they exhibit accessibility at their promoter or potential nearby regulatory regions but are not expressed in Mesothelial cells (**Figures 3E, F**). This finding suggests that chromatin at a subset of *Procr-like* Mesothelial^Upk3b-low^ cells might be primed for activation, thus allowing eventual expression of genes associated with BP/ductal (e.g. *Elf5*, *Adamts16*, *Bicc1*) or EP/endocrine (*Ncam1*, *Syt13*, *Runx1t1*) lineage commitment (43, 48, 56–59), in agreement with the developmental trajectory inferred from our integrated scRNA-seq analysis (**Figure 2E**). The genes with accessible chromatin and expression consistently enriched in mesothelial cells included *Igfbp4*, previously associated with cell proliferation and IGF1 signaling (60).

Given the uncertainty in the association of distal regulatory regions to their regulated genes, the analysis presented above was restricted to accessible regions located within or nearby gene bodies, thus increasing the accuracy of the associations. As a complementary approach, we performed a *de novo* motif analysis on all high confidence (*p adj.*<0.005, average log_2_ fold change>1) Mesothelial^Upk3b-low^ cluster marker regions. The results revealed a significant enrichment for 28 DNA binding motifs. Interestingly, the top enriched sequence was a bHLH motif that matched with extremely high similarity (HOMER Score>0.9) to either Ptf1a, Twist2 or Ascl1, among others, suggesting that these regions could be bound by transcription factors that are expressed in pancreatic epithelial progenitors (Ptf1a), mesenchymal (Twist2) or neural-crest-derived/Schwann cells (Ascl1, **Figure 3G**). While neither of these factors is expressed in mesothelial cells from our integrated scRNA-seq analysis (**Supplementary Figure 3F**), we found that these cells expressed other suitable candidates whose recognition sequence also matched this bHLH motif with high affinity (e.g. *Tcf3*, *Tcf12*, **Figure 3G**, **Supplementary Figure 3F**). However, expression of these factors could be also detected in both pancreatic epithelial and mesenchymal cells (red and blue boxes in **Supplementary Figure 3G**). On the other hand, consistently with its potential activation, we found mild accessible chromatin enrichment at the *Ptf1a* promoter and some of its distal regulatory regions in Mesothelial^Upk3b-low^ cells (red boxes in **Figure 3H**). Of note, these regions present modest accessibility in progenitor cells (Duct/Acinar, Duct/BP), which is importantly increased in Acinar cells.

Other highly enriched motifs matched those recognized by Nfi (Nfix, Nfia, Nfib, Nfic), Tead and Rbpj transcription factors that, despite playing relevant roles in epithelial pancreatic progenitors (44, 61–63), are also expressed in mesenchymal/mesothelial cells (**Figure 3G**, **Supplementary Figures 3F, G**). Noteworthy, *Nfib* expression is selectively shared among mesothelial and BP/ductal cells, and it is downregulated in EP upon commitment to the endocrine lineage (**Figure 3G**). Conversely, *Tead2* is expressed in mesothelial cells and selectively upregulated in BP/ductal cells, its expression peaks in EP cells to be then silenced from the Fev^+^ stage and on in endocrinogenesis (**Figure 3G**). We have previously shown that TEAD and YAP are key components of the enhancer network in pancreatic progenitors (62), and this signaling axis finely tunes progenitor cell commitment along the endocrine fate (64, 65). Tead motif enrichment in mesothelial cells is consistent with a progenitor role for these cells. Thus, *Nfib* and *Tead2* transcription factors support the developmental trajectory inferred from out integrated scRNA-seq analysis between mesothelial and BP/ductal cells (**Figure 2E**).

Interestingly we found the Hand2 DNA recognition sequence enriched among the top scoring motifs, and the gene encoding for this TF is expressed in mesothelial cells of the embryonic pancreas (**Figure 3G**). Consistently with our analyses so far, *Hand2*, as well as most factors expressed in mesothelial cells, are also expressed in adult Procr^+^ progenitors of the original dataset (**Supplementary Figure 3H**). *Hand2*, in particular, is also expressed in pancreatic stellate and mesenchymal cells (**Supplementary Figures 3G, H**). Noteworthy, this factor has previously described functions in mesenchymal/mesothelial, neuronal and cardiac development (66).

Taken together, these results identified transcription factors with a shared expression profile supporting a mesothelial-ductal/BP lineage relationship, as well as other factors with genomic regulatory regions that could be primed for activation in *Procr-like* mesothelial cells.

### 3.4 Islet-like organoids differentiated *in vitro* from adult Procr^+^ progenitors mimic the transcriptional profile of the mesothelial and ductal/BP clusters in mouse pancreas development

Our results suggest that the originally described adult Procr^+^ progenitors globally share its transcriptional profile with mesothelial cells. This includes the expression of genes coding for transcription factors (e.g. *Sox4*, *Sox11*, *Nfib*, *Tead2*, *Rbpj*) and other regulators (e.g. *Bicc1*, *Spp1*, *Runx1t1*) that play important roles in epithelial pancreatic progenitors (**Figures 2F**, **3F, G**). Additionally, a subset of these present accessible chromatin at genes coding for transcription factors with crucial roles in epithelial pancreatic progenitors (e.g. *Ptf1a*, *Pdx1*, **Figure 3H**, **Supplementary Figure 3C**). These findings, combined with a developmental trajectory analysis inferred from the scRNA-seq data (**Figure 2E**), suggest that adult Procr^+^ progenitors could engage in endocrinogenesis through a ductal/BP intermediate stage. To further explore this possibility, we interrogated the global expression profile of adult Procr^+^ progenitors co-cultured *in vitro* with endothelial cells and differentiated into islet-like organoids, which contained functional β-cells after 28 days of culture (scRNA-seq data kindly provided by the authors) (22).

**Figure 4:**
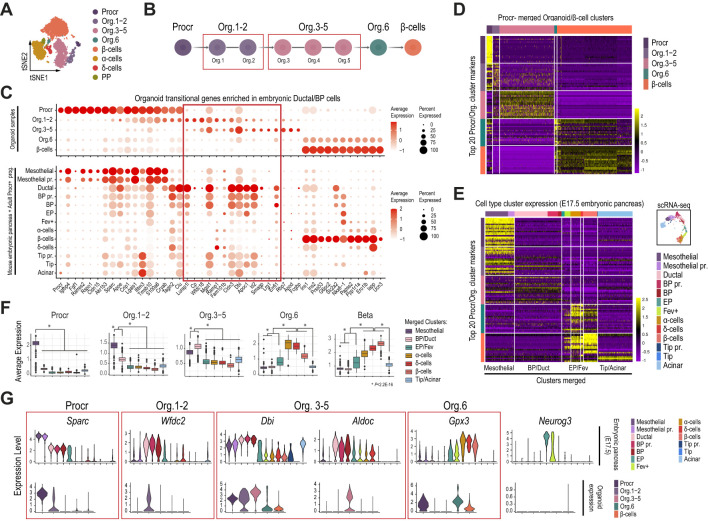
Islet-like organoids differentiated *in vitro* from adult Procr^+^ progenitors mimic the transcriptional profile of the mesothelial and ductal/BP clusters in mouse pancreas development. **(A)** tSNE plot of organoid single-cell transcriptomes differentiated *in vitro* as originally described (22). **(B)** Schematic depicting organoid sample merging for downstream analyses. **(C)** Dot plots showing the expression of a selected gene subset previously reported to be sequentially activated upon Procr^+^ progenitor commitment to the β-cell lineage. Gene expression is shown for cell clusters of the organoid dataset (top), and the pancreatic epithelial and mesothelial cells of the mouse embryonic pancreas (bottom) as clustered in **Figure 2A**. **(D)** Heatmap showing the scaled gene expression values for the top 20 cluster marker genes recovered from the Procr organoid analysis. Gene expression data corresponding to Procr, organoid stages and β-cells from the organoid dataset clustered as in **(A)**. **(E)** Heatmap showing the scaled gene expression values for the top 20 cluster marker genes recovered from the Procr organoid analysis (same subset as in **D**). Gene expression data corresponding to pancreatic epithelial and mesothelial cells of the E17.5 mouse embryonic pancreas clustered as in **Figure 3A**. **(F)** Boxplots depicting the average expression for the top 20 organoid cluster markers in cells of the E17.5 mouse embryonic pancreas, clustered and labeled as in **Figure 3A**. Based on the shared expression patterns in this analysis, cell clusters were further merged into broader categories as indicated in **(E)** and described next: Mesothelial (including Mesothelial and Mesothelial pr. cells), Duct/BP (Ductal, BP, and BP pr.), EP/Fev (EP and Fev^+^ cells) and Tip/acinar (Tip, Tip pr. and acinar cells). The *P*-value was calculated with the Wilcoxon rank-sum test. **(G)** Violin plots depicting the expression of selected Procr, Org. 1-2, Org. 3-5 and Org. 6 top marker genes for the pancreatic epithelial and mesothelial cells of the E17.5 mouse embryonic pancreas (top), clustered as in **Figure 3A**, and for the different Procr, organoid and β-cell stages (bottom) clustered from the organoid dataset as in **(A)**.

The original report identified six intermediate organoid stages (Org.1-6) between Procr^+^ progenitors and β-cells (22). For our downstream analyses, we merged organoid stages 1 and 2 (Org. 1-2), and stages 3 to 5 (Org.3-5), due to their closely related global transcriptional profiles (**Figures 4A, B**). We initially evaluated the expression of a selected gene subset previously reported to be sequentially activated upon Procr^+^ progenitor commitment to the β-cell lineage (**Figure 4C**, top dotplot). Notably, most of the transitional genes that were switched off in Procr^+^ progenitors and activated in the Org1-2 and Org3-5 intermediate stages were also highly and selectively expressed in Ductal and BP cells of the mouse embryonic pancreas (**Figure 4C**, bottom dotplot and red box). Supporting their priming for activation, several of these genes displayed accessible profiles (summarized as gene activity scores) in mesothelial cells of the E17.5 mouse embryonic pancreas (**Supplementary Figures 4A, B**). Additionally, supporting their link between Mesothelial^Upk3b-high^ and ductal/BP cells, Mesothelial^Upk3b-low^ cells exhibited increased gene activity scores for most of these genes.

To further extend these findings, we next focused the analysis on the initial timepoint of the previously reported *in vitro* differentiation protocol (day 7), which contained the largest number of intermediate organoid stages, as well as a small fraction of endocrine cells (22). As expected, the top 20 cluster marker genes for each organoid stage revealed a highly selective expression pattern (**Figure 4D**). Thus, we defined sets of 20 genes that were highly enriched in Procr^+^ progenitors, Org.1-2, Org. 3-5, Org.6 and β-cells. Globally, these genes displayed a transitional expression pattern among consecutive stages. We next examined whether these set of genes were expressed in the pancreatic progenitor and differentiated cell clusters identified in our previous analysis for the E17.5 mouse embryonic pancreas, the stage at which we detected mesothelial cells with the transcriptional profile most closely resembling adult Procr^+^ progenitors. As expected, Procr^+^ markers from the organoid study were expressed at higher levels in Mesothelial and Mesothelial pr. clusters profiled from the E17.5 pancreas (**Figure 4E**). Strikingly, Org. 1-2, Org. 3-5 and Org. 6 cluster markers exhibited a decreasing expression pattern, on average, in mesothelial clusters, and a gradually increasing expression pattern sequentially in BP and ductal (BP/Duct) cells, EP and Fev^+^ (EP/Fev), α-, δ- and β-cell clusters of the embryonic pancreas (**Figures 4E, F**). Illustrative examples following this transitional trend include: 1) *Sparc*, an adult Procr^+^ progenitor marker that is highly expressed in mesothelial cells of the mouse embryonic pancreas, gradually downregulated in ductal, BP, EP, and finally silenced in Fev^+^ and endocrine cells; 2) *Wfdc2* and *Dbi*, Org. 1-2 and Org. 3-5 markers that are upregulated in ductal/BP and EP cells; and 3) *Gpx3*, an Org. 6 marker that is upregulated in Fev^+^ cells and expressed at higher levels in α-, δ- and β-cells (**Figure 4G**).

Collectively, these findings suggest that *in vitro* differentiation of adult Procr^+^ progenitors (co-cultured with endothelial cells as described previously) (22) recapitulates a transcriptional pattern that mimics the sequential activation of at least some BP/ductal lineage enriched genes, whose downregulation is followed by expression of EP/Fev, α and δ cell markers, and finally β-cell genes in cells of the mouse embryonic pancreas. These genes, however, do not include *Neurog3* (**Figure 4G**).

### 3.5 *Procr-like* mesothelial cells are also identified in the human embryonic pancreas

We have previously reported that a Procr-like subpopulation was spontaneously specified from human induced pluripotent stem cells (iPSCs) differentiated *in vitro* to pancreatic progenitor cells (67). To gain further insights into whether mesothelial cells with a Procr-like transcriptional profile, as identified in the mouse embryonic pancreas, have counterparts in the developing human organ, we integrated the mouse datasets used in the analyses described above with recently reported scRNA-seq data from the 4 to 11 weeks post-conception human pancreas (W4–11) (**Supplementary Figure 5A, C**) (68). An initial analysis on the human samples, grouped by stages W4-6 and W7-11 as originally reported, identified mesenchymal (*COL3A1* ^+^), endothelial (*PECAM1* ^+^), immune (*RAC2* ^+^), erythrocytes (*HBA1* ^+^), neural crest-derived/Schwann cells (*SOX10* ^+^), pancreatic epithelial cells (*EPCAM* ^+^; red boxes in **Supplementary Figure 5B, D**) as well as mesothelial cell clusters with Procr-like expression profile in both groups of samples (*PROCR^+^*, *UPK3B^+^*, *IGFBP5* ^+^; purple boxes in **Supplementary Figure 5B, D**). We also found cells with a transcriptional profile corresponding to liver progenitors (*AFP^+^*) in the human embryonic datasets, as described (68).

**Figure 5:**
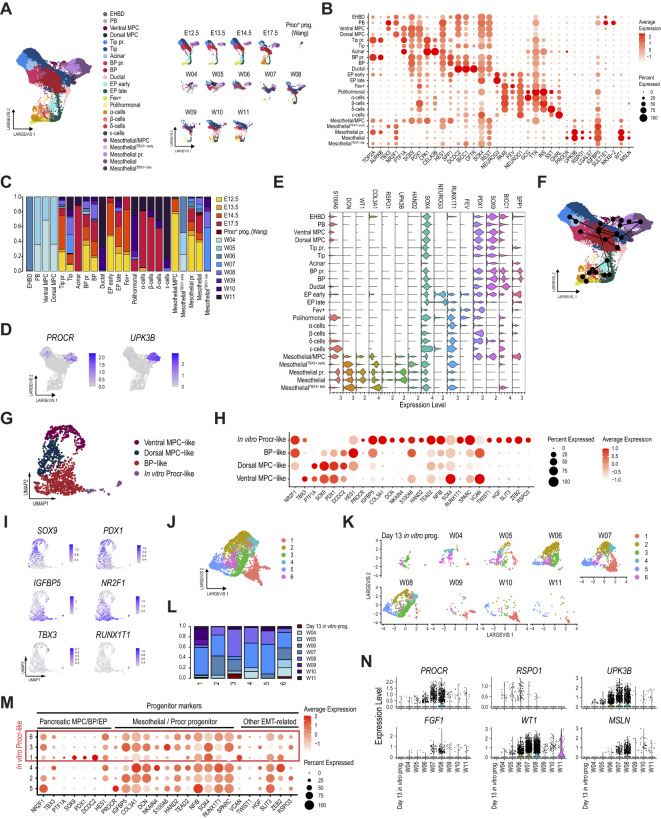
*Procr-like* mesothelial cells are also identified in the human embryonic pancreas. **(A)** Dimension plot of integrated single-cell pancreatic epithelial and mesothelial transcriptomes profiled from the E12.5-E17.5 mouse embryonic pancreas, the W4-W11 human embryonic pancreas, and Procr^+^ progenitors profiled from mouse adult islets. Dimension plots in the right panel show cell distribution according to sample timepoint. **(B)** Dot plot showing the expression of selected pancreatic, mesothelial, and Procr^+^ progenitor markers used to match cell clusters. Cells clustered as in **(A)**. **(C)** Proportion of cells from each dataset contributing to the cell clusters identified in **(A)**. **(D)** Feature plots showing the expression of *PROCR* and *UPK3B*. **(E)** Violin plots showing the expression of selected pancreatic and Procr^+^ progenitor markers. **(F)** Trajectory analysis ordering of mesothelial and pancreatic epithelial cells clustered as shown in **(A)**. **(G)** UMAP plot of single-cell transcriptomes profiled from *in vitro* differentiated day 13 pancreatic progenitors. **(H)** Dot plot showing the expression of selected well-known pancreatic epithelial and mesothelial/Procr^+^ progenitor markers used to match cell clusters with their closest progenitor cell type. Additional cluster markers are included to highlight genes with potential roles in EMT. **(I)** Feature plots showing expression of selected pancreatic multipotent progenitor markers (*SOX9*, *PDX1*, *NR2F1*, *TBX3*), the mesothelial/Procr^+^ marker *IGFBP5*, and the NGN3 target gene *RUNX1T1*. **(J)** Dimension plot showing re clustering of human *in vitro* Procr-like cells from **(G)**, and human cells of mesothelial clusters from **(A)**. **(K)** Dimension plots showing the cell distribution according to sample of origin. **(L)** Proportion of cells from each dataset contributing to the cell clusters identified in **(J)**. **(M)** Dot plot showing the expression of selected well-known pancreatic epithelial, mesothelial/Procr^+^ progenitor, and other EMT-related genes. **(N)** Violin plots showing the expression of key Procr^+^ progenitor and mesothelial cell markers in mesothelial cells analyzed in panel **(J)**, split by dataset.

We next integrated the pancreatic epithelial cells and those clusters matching the Procr^+^ progenitor transcriptional signature from the embryonic mouse and human pancreas datasets to interrogate in more detail their potential lineage relationship. This analysis allowed discriminating the same cell subpopulations described above for the mouse embryonic pancreas, as well as the early-stage-specific human progenitor cell populations reported in the original analysis (68), corresponding to dorsal (*PDX1* ^+^, *SOX9* ^+^ *PTF1A* ^+^, *NR2F1* ^+^) and ventral (*PDX1* ^+^, *SOX9* ^+^ *PTF1A* ^+^, *TBX3* ^+^) pancreatic multipotent progenitor cells (MPC), pancreato-biliary (PB) progenitors (*NKX6.2* ^+^, *SULT1E1* ^+^) and extrahepatic bile ducts (EHBD, *SPP1* ^+^, *SULT1E1* ^+^; **Figures 5A, B**, **Supplementary Table 6**). We also found two discrete EP (*NEUROG3* ^+^) populations, the first one predominantly present at early stages (EP^early^, E12.5-E14.5) in the mouse datasets, and the second one was present in both species (EP^late^, **Figure 5C**). The latter one subsisted in the mouse until E17.5. As before, we tracked “Mesothelial” and “Mesothelial proliferative” cell clusters that co-expressed Procr^+^ progenitor markers (*PROCR*, *RSPO1*, *UPK3B, IGFBP5)* and were additionally subset upon the co-expression of proliferative genes (*TOP2A* ^+^, *AURKB* ^+^) (**Figures 5B, D**). Notably, *PROCR* expression was detected in the Mesothelial cell clusters, as well as in Tip and Acinar cells, with lower levels observed in a small fraction of BP and Ductal cells (**Supplementary Figure 5F**). Consistent with our previous findings, Procr^+^ progenitors from adult pancreatic islets mapped to the Mesothelial cluster, which included cells from both species and most pancreatic embryonic stages (**Figures 5A, C**). Thus, the previously reported adult Procr^+^ progenitor population (22) presents a global transcriptional profile that also matches in humans a subset of cells previously associated with mesothelial cells (23, 40, 68).

Interestingly, this integrated analysis identified three additional mesothelial populations that were separately clustered. Two of these populations were characterized by the co-expression of Procr^+^ progenitor markers (*PROCR*, *UPK3B*, *IGFBP5*) at lower levels, along with the mesothelial marker *WT1* and the early-stage human MPC markers *NR2F1* and *TBX3* (**Figure 5B**). These populations are hereafter referred to as Mesothelial^TBX3+ early^ and Mesothelial^TBX3+ late^, according to the developmental stages in which they are found (**Figures 5A–C**). Notably, these were exclusively present in the human samples. Unexpectedly, the third population co-expressed low levels of the Procr^+^ progenitor markers along with the mesothelial marker *WT1* and the MPC/Tip markers *PTF1A*, *SOX9* and *PDX1* (Mesothelial/MPC). Of note, although this third population was almost exclusively composed of mouse cells, this cluster was not detected in our previous scRNA-seq analysis. This suggests that the broader transcriptomic contextualization provided by this new analysis allowed cells to be grouped differently, revealing a hybrid transcriptional profile that particularly matched the Mesothelial^Upk3b-low^ cell cluster identified in our scATAC-seq analysis (**Figures 3B**). Interestingly, the location of the EP^early^ population in the dimensional plot suggests that it could represent an intermediate progenitor stage between Mesothelial/MPC cells and α-cells during early developmental stages in mice (**Figure 5A**, right panel), suggesting that the former may act as facultative progenitors during the first transition. Additionally, while all mesothelial populations identified in this analysis expressed the mesothelial marker *WT1*, the expression of *MSLN*, another characteristic mesothelial marker, was not detected in the three novel mesothelial cell clusters (**Figure 5B**). Intriguingly, all mesothelial populations also shared the expression of *NR2F1* with dorsal MPC, PB and EHBD, while *TBX3* was selectively detected in Mesothelial^TBX3+ early^, Mesothelial^TBX3+ late^, Ventral MPC, PB and EHBD. Consistent with its expression pattern in pancreatic progenitors, mesothelial cells also expressed *REST*.

The originally reported mouse adult Procr^+^ progenitors are also transcriptionally similar to *Procr-like* mesothelial cluster cells present in the human developing pancreas. Notably, while most mesothelial clusters did not directly overlap with pancreatic epithelial cells in the dimension plot, Mesothelial^TBX3+ late^ cells linked the mesothelial clusters with tip and BP cells (**Supplementary Figures 5E, G**). Consistently, a developmental trajectory revealed a progression from Mesothelial^TBX3+ early^ through Mesothelial and Mesothelial^TBX3+ late^ to tip and BP cells, suggesting a lineage relationship in endocrinogenesis within the developing human pancreas (**Figure 5F**). Unlike our previous analysis, this broader contextualization further discriminated mesothelial cells into two groups: those with higher expression levels of Procr^+^ progenitor markers, such as *UPK3B* and *RSPO1*, and low or undetectable expression of ductal and BP marker genes, such as *SPP1*, *BICC1*, and *SOX9* (Mesothelial and Mesothelial pr.); and those with lower expression of Procr^+^ progenitor markers but higher expression of pancreatic progenitor markers, such as *PDX1*, *SOX9* (Mesothelial/MPC), *BICC1*, and *RUNX1T1* (Mesothelial^TBX3+ early^ and Mesothelial^TBX3+ late^; **Figure 5E**).

Strikingly, Mesothelial^TBX3+ early^ cells also co-expressed high levels of *HAND2*, which binds one of the most enriched DNA binding motifs recovered from Mesothelial^Upk3b-low^ cells in our scATAC-seq analysis. We also observed a complementary expression pattern for the levels of *COL3A1*, *UPK3B*, *RSPO1*, *SOX4*, *RUNX1T1* in mesothelial clusters. Conversely, other Procr^+^ progenitor markers presented an increasing expression trend (e.g. *DCN*; **Figure 5E**). These observations, along with the use of a state-of-the-art tool for doublet removal, argue against the possibility that mesothelial clusters co-expressing pancreatic progenitor and Procr^+^ progenitor markers result from sequencing doublets, as a hypothetical combination of mesothelial and pancreatic progenitors would hardly account for the complementary expression patterns described above. Resolving this discrepancy will certainly require future experimental validation.

Unexpectedly, a cell population partially matching the *Procr-like* mesothelial transcriptional profile is spontaneously co-specified in human iPSCs differentiated into pancreatic progenitors. Cells in this protocol are gradually guided through definitive endoderm (with high efficiency), followed by a foregut progenitor stage, and finally into the pancreatic lineage (62, 67). This finding argues against these Procr-like cells having a mesodermal origin, as would be expected for mesothelial cells (69).

To explore this in more detail, we assessed the transcriptional profile of Procr-like cells co-specified *in vitro* from human iPSCs differentiated to multipotent pancreatic progenitors (MPC) at Day 13 of the differentiation protocol (67). This analysis revealed four clusters, three of which were named based on the expression of known pancreatic progenitor markers: dorsal MPC-like (*PDX1* ^+^, *SOX9* ^+^ *PTF1A* ^+^, *NR2F1* ^+^), ventral MPC-like (*PDX1* ^+^, *SOX9* ^+^ *PTF1A* ^+^, *TBX3* ^+^) and BP-like (*PDX1* ^+^, *SOX9* ^+^, *DCDC2* ^+^, *HES1* ^high^, *PTF1A-*; **Figures 5G–I**, **Supplementary Table S7**). Additionally, we identified a fourth cluster closely resembling the transcriptional profile of Procr^+^ progenitors cells (*PROCR^+^*, *IGFBP5^+^*, *COL3A1^+^, DCN^+^, NKAIN4^+^ (* 22); referred to hereafter as *in vitro* Procr-like) (**Figure 5H**). Notably, *in vitro* Procr-like cells also expressed high levels of most of the novel markers introduced above for embryonic *Procr-like* mesothelial cells, including *HAND2*, *TEAD2*, *NFIB*, *RUNX1T1* and *SPARC*. Unexpectedly, we also detected the expression of other interesting markers that may underlie the transition between epithelial and mesenchymal characteristics, including Versican (*VCAN*), *TWIST1*, *HGF*, *SLIT3*, *ZEB2* and *RSPO3* (**Figure 5H**) (24, 70–74).

An in depth comparison was next performed by reintegrating cells from all the mesothelial clusters corresponding to the human embryonic pancreas with *in vitro* Procr-like cells to reveal similarities in their expression profiles (**Figure 5J**). Strikingly, *in vitro* Procr-like cells clustered together with specific subsets of human embryonic mesothelial cells overrepresented in W4-8 samples (**Figures 5K, L**). Importantly, this developmental stage matches the approximate differentiation timepoint of the human iPSC differentiation protocol. Surprisingly, despite the clusters matching *in vitro* Procr-like cells did share expression of *PROCR* and several other adult mouse Procr^+^ progenitor markers, the expression of the mesothelial-associated markers *UPK3B*, *RSPO1* and *FGF1* could not be detected (**Figures 5M, N**). These cells did not express other classic mesothelial markers such as *WT1* and *MSLN*, either, consistent with the low/undetectable expression for these markers in Procr-like cells of the W4 human embryonic pancreas. This unexpected finding suggests that a Procr-like cell population that is negative for mesothelial markers shares a lineage relationship with pancreatic progenitors.

Taken together, these analyses show that a mesothelial cell population with a Procr-like transcriptional profile, potentially lineage-related to pancreatic progenitors, exists in the human developing pancreas, and this unexpected developmental trajectory is recapitulated *in vitro*. Based on their clustering with mesothelial cells from the W4–8 human embryonic pancreas (**Figures 5K, L**), their shared expression profile of *HAND2* (**Figures 5E, H**), and the low or undetectable expression levels of other markers such as *WT1*, *MSLN*, and *UPK3B* (**Figure 5N**), our findings support that *in vitro* Procr-like cells closely resemble the Mesothelial^TBX3+ early^ population identified in the human embryonic pancreas. Although our combined transcriptional and epigenomic analyses strongly suggest that these cells are related to BP/ductal cells, further research is needed to confirm the hypotheses presented in this study.

## 4 Discussion

The existence of facultative progenitors in the adult pancreas has been debated for decades, with early studies suggesting their location within the ductal tree (6). Recent findings continue to support this hypothesis (7–17), although the only widely accepted source of new insulin-producing cells involves α-, δ-, and γ-cell interconversion under specific conditions (18–21).

A recent study identified a novel progenitor population in adult mouse pancreatic islets capable of regenerating endocrine cell types (22). Characterized by scRNA-seq, this population expresses epithelial (*Epcam*, *Cldn10*), mesenchymal (*Vim*, *Col3a1*), and progenitor markers (*Procr*, *Rspo1*, *Upk3b*). However, there have been no further reports to substantiate these novel findings, and additional studies will be critical to validate these observations.

Leveraging the scRNA-seq datasets from the original report, we sought to identify cell types that align with the transcriptional profile of these adult Procr^+^ progenitors. Notably, this specific profile was absent in other mouse and human pancreatic islet datasets. A plausible explanation lies in the experimental design of the original study, which profiled a mixture of islet and non-islet cells (22), potentially capturing signals from previously described progenitor populations, such as centroacinar or ductal cells (4).

Unexpectedly, our analysis revealed a transcriptionally similar population in the embryonic pancreas of both mice and humans. Moreover, the transcriptional profile of adult Procr^+^ progenitors closely resembled that of mesothelial cells (23, 24, 56). In integrated analyses, these progenitors consistently clustered with a subpopulation of mesothelial cells, suggesting lineage-related connections. Despite the fact that mesenchymal cells of mesothelial origin are known to play a key role during the early stages of pancreas specification and development (75), their direct contribution to the epithelial pancreatic progenitor lineage remains elusive. It is known that mesothelial cells contribute to the pancreatic stellate cell lineage during organ development, and these cells play important roles in tissue repair and regeneration (69, 76). Interestingly, previous reports suggest that pancreatic stellate cells can act as facultative pancreatic progenitors (77, 78).

By comparing gene expression profiles of Procr^+^ progenitors, mesothelial cells, and intermediate progenitor stages involved in β-cell development, we identified genes potentially linking *Procr-like* mesothelial cells to epithelial progenitors during pancreas development. This comprehensive “dictionary” of genes serves as a valuable resource for investigating the mechanisms underlying β-cell regeneration in adult tissues, particularly those involving ductal or acinar cells.

Cells with a Procr-like transcriptional profile are more closely aligned with mesothelial cells than pancreatic epithelial cells. Upon detailed reanalysis of these, alongside pancreatic epithelial cells, we uncovered a transcriptionally interconnected trajectory, suggesting that at least a subset of *Procr-like* mesothelial cells may be lineage-related to epithelial progenitors.

Our results suggest a developmental lineage relationship: BP → duct → Mesothelial pr. → Mesothelial cells, with the latter being overrepresented in late-stage (E17.5) mouse embryonic samples and exhibiting the closest transcriptional profile to adult Procr^+^ progenitors. Conversely, adult Procr^+^ progenitors differentiated *in vitro*, along with endothelial cells, appear to partially reverse this lineage relationship: Procr^+^ progenitors → duct/BP-like cells → endocrine cells. Thus, the ductal/BP stage serves as a crossroads between Procr^+^ progenitors and endocrine cells. Notably, this lineage relationship does not involve the expression of *Neurog3*, as during development BP/duct cells are directly related to Mesothelial proliferative cells (with *Neurog3* not detected at any of these stages), and the same holds true for *in vitro* differentiated adult Procr^+^ progenitors. However, we cannot entirely exclude the possibility that *Neurog3* is expressed at very low levels at any point during these transitions, potentially below the single-cell detection threshold.

Further supporting their endocrine competence, we report that a subset of *Procr-like* mesothelial cells (Mesothelial^Upk3b-low^ cells identified in our scATAC-seq analysis) exhibit chromatin accessibility at several key regulators of epithelial pancreas development and endocrinogenesis. Notably, these regulators are either not expressed or barely detected in mesothelial cells, suggesting that their associated regulatory regions may be primed for activation. These regulators include *Runx1t1*—a Ngn3-regulated transcriptional co- repressor (26); *Syt13*—recently characterized as a critical regulator of endocrine cell egression and islet formation (43); and *Pdx1* and *Ptf1a*, two essential early-stage pancreatic regulators (44), among others.

The role of *Upk3b* in epithelial progenitors is particularly intriguing. Highly expressed in mesothelial cells lacking classical pancreatic markers (*Pdx1*, *Ptf1a*, *Sox9*), its expression diminishes in transitional cells with shared pancreatic and mesothelial features (i.e. Mesothelial^TBX3+ early^, Mesothelial^TBX3+ late^, Mesothelial/MPC). Interestingly, recent findings indicate that *Upk3bl1*, an important paralog for *Upk3b*, is upregulated in EP at advanced stages of endocrinogenesis (43). We have also previously reported that *Upk3a*, another paralog for *Upk3b*, is expressed in adult α-cells and it is downregulated upon massive β-cell ablation (79), a process that spontaneously triggers the α- to β-cell conversion process.

Notably, genes encoding transcription factors with relevant roles in pancreatic progenitors, such as *Bicc1*, *Sox4*, *Nfib*, and *Tead2*, are also expressed in mesothelial and mesenchymal cells and may facilitate transitions between *Procr-like* mesothelial cells and ductal/BP precursors. Consistently, we found that DNA-binding motifs enriched at chromatin-accessible marker regions of the Mesothelial^Upk3b-low^ scATAC-seq cluster are often either ambiguously bound by transcription factors specific to the pancreatic epithelial lineage (e.g. Ptf1a) or mesenchymal/mesothelial lineage (e.g. Twist1/2), or bound by factors commonly expressed in both lineages (e.g. Nfib, Tead2). Additionally, Hand2, expressed in both mesothelial cells and adult Procr+ progenitors, emerges as a potential regulator of facultative pancreatic progenitors.

We also identified *Procr-like* mesothelial cell populations in the human embryonic pancreas, equivalent to those in mice. Additionally, we found two unique subpopulations, Mesothelial^TBX3+ early^ and Mesothelial^TBX3+ late^, exclusive to humans. Both co-express *TBX3* and *NR2F1*, markers of early ventral and dorsal MPCs. Mesothelial^TBX3+ early^ cells are transcriptionally linked to early-stage MPCs, while Mesothelial^TBX3+ late^ cells are closer to BP/ductal cells. Our trajectory analysis revealed a progression from Mesothelial^TBX3+ early^ through Mesothelial and Mesothelial^TBX3+ late^ to tip and BP cells, suggesting a lineage relationship in endocrinogenesis within the developing human pancreas.

Despite using advanced tools to eliminate potential doublets in the scRNA-seq and scATAC-seq datasets analyzed (53), we cannot fully exclude the possibility that the observed transcriptional lineage relationships—marked by clusters co-expressing pancreatic progenitor markers (*Pdx1*, *Sox9*, *Ptf1a*) and mesothelial markers (*Msln*, *Wt1*, *Upk3b*, *Col3a1*)—stem from unresolved doublets. However, these clusters were consistently detected in scRNA-seq datasets from both mouse and human embryonic pancreas samples across multiple developmental stages, as well as in independent scATAC-seq datasets.

Further supporting the existence of mesothelial cells with Procr-like characteristics and their lineage relationship with MPCs, we describe here that a small subpopulation with a Procr-like signature (*in vitro* Procr-like) is spontaneously co-specified in human iPSCs directed along the pancreatic progenitor lineage. Notably, the initial stages of this protocol involve the derivation of definitive endoderm cells with high efficiency, followed by specification to the subsequent foregut progenitor stage. Since mesothelial cells (found here to partially share the Procr+ progenitor transcriptional profile) are specified from the mesoderm lineage, it is difficult to conceive that the cells identified in the *in vitro* differentiation protocol are of mesothelial origin. Instead, this suggests the possibility of a direct lineage relationship between epithelial pancreatic progenitors and mesothelial-like progenitor cells. Rather than suggesting a bias from the *in vitro* conditions, we interpret these findings as evidence that the *in vitro* differentiation system is partially recapitulating a Procr-like progenitor population. This population is potentially aligned with specific Procr-like mesothelial cells in the human embryonic pancreas, as evidenced by the unbiased clustering of these progenitor cell populations (**Figures 5J–L**). This suggests that the *in vitro* model is capturing key aspects of the *in vivo* context, although further validation is required to fully confirm this alignment.

We propose that progenitor cells undergoing EMT during pancreas development—facilitating migration from the trunk/ductal tree—may align transcriptionally with mesenchymal/mesothelial cells. Supporting this, an endocrine progenitor substage (EP2) expressing mesenchymal markers (*Col3a1*, *Vim*, *Twist1*, *Zeb2*, *Snai1*) but almost undetectable levels of pancreatic progenitor markers (*Pdx1*, *Sox9*, *Hnf1b*, *Foxa2*, *Neurod1*) has been reported (24, 25). Interestingly, this profile resembles human Procr-like cells identified *in vitro* during pancreatic differentiation using human iPSCs.

Taken together, our results suggest that a subset of mesothelial-like cells closely resembling the adult Procr^+^ progenitor transcriptomic profile are physiologically specified during mouse and human pancreas development. Furthermore, epigenomic analyses support their epithelial pancreatic progenitor competence to differentiate into the endocrine lineage. Despite originally mapped to the adult mouse pancreatic islets, our results suggest that they could be tracked within the pancreatic ductal structures, as *Procr-like* mesothelial cells are transcriptionally closer to ductal cells. Future work is expected to yield light into whether any of the several potential progenitor cell sources recently reported (7–10), could match the embryonic mesothelial or adult Procr^+^ progenitor cell populations.

## Data Availability

The scRNA-seq data analyzed in this study is publicly available from the GEO (https://www.ncbi.nlm.nih.gov/geo/), SRA (https://www.ncbi.nlm.nih.gov/sra), GSA (https://ngdc.cncb.ac.cn/gsa/) or NODE (https://www.biosino.org/node/) repositories, with the reference numbers specified in Methods.
