# Supplementary material for: Identification of mouse and human embryonic pancreatic cells with adult Procr+ progenitor transcriptomic and epigenomic characteristics
Source: Front Endocrinol (Lausanne). 2025 Feb 13;16:1543960. doi: 10.3389/fendo.2025.1543960 (PMC11864936; doi:10.3389/fendo.2025.1543960)
Supplement: Supplementary file 1 [file DataSheet1.docx]

Supplementary Material

**Identification of mouse and human embryonic pancreatic cells with adult Procr^+^ progenitor transcriptomic and epigenomic characteristics**

**Authors**

Ana C. Heidenreich,^1,2^ Lucas Bacigalupo,^1,2^ Martina Rossotti,^1,2^ Santiago A. Rodríguez-Seguí^1,2†^

**Affiliations**

^1^ Instituto de Fisiología, Biología Molecular y Neurociencias (IFIBYNE-UBA-CONICET), Universidad de Buenos Aires, Facultad de Ciencias Exactas y Naturales, Buenos Aires, [C1428EGA], Argentina.

^2^ Departamento de Fisiología, Biología Molecular y Celular, Universidad de Buenos Aires, Facultad de Ciencias Exactas y Naturales, Ciudad Universitaria, Buenos Aires, [C1428EGA], Argentina.

^†^ Corresponding author: Santiago A. Rodríguez-Seguí, [srodriguez@fbmc.fcen.uba.ar](mailto:srodriguez@fbmc.fcen.uba.ar)

**Supplementary methods**

**Single-cell RNA-seq Data Processing and Analysis**

All analyses were performed using R version 4.1.1. The single-cell gene expression profiles were processed with Seurat (1) or Conos (2) as described in each section.

Mouse Adult Pancreatic Islet Data Preprocessing. Publicly available raw 10X Genomics FASTQ files for the Chung et al. (3), De Jesus et al. (4), Tritschler et al. (5), and Kleiber et al. (6) datasets (SRA accessions SRR10096826, SRR14119316, SRR8754575, and SRR11866759, respectively) were aligned to the mm10 mouse genome using CellRanger (v3.0.2). Already aligned, quantified gene expression data generated using the CEL-Seq protocol for the Baron et al. (7) dataset were downloaded from the Gene Expression Omnibus (GEO) (accession GSM2230761). Finally, the Wang et al. dataset, which contains the original mouse pancreatic islet cells already clustered, including the Procr^+^ progenitor population, was kindly provided by the authors (8). It includes 3,706 cells and 20,547 genes, clustered as reported into endocrine cells (β, α, δ, PP), endothelial, stromal, stellate, acinar, ductal, immune, and the novel Procr^+^ population (130 cells).

Unless otherwise specified, all six samples were equally processed as follows: For the Chung et al. (3), De Jesus et al. (4), Tritschler et al. (5), and Kleiber et al. (6) datasets, CellRanger output data were imported into Seurat using the load10X function. SoupX was then applied to remove ambient RNA contamination using the autoEstCont and adjustCounts functions. For the Baron et al. dataset (7), a Seurat object was created using the counts data available from GEO. SoupX was not applied to this sample for RNA removal since this dataset was sequenced using the CEL-Seq protocol, and SoupX is optimized for 10X Genomics dataset processing. For the Wang et al dataset, the already quantified and clustered Seurat object was loaded. Since raw data for this sample were not re-quantified, SoupX procesing was not applied either.

Cells with unique feature counts between the 10^th^ and 90^th^ percentiles for each sample, and mitochondrial percentages below 10% were retained. SCTransform (for Seurat integration) or NormalizeData (for Conos integration) were applied for normalization. Potential doublets were identified using scDbIFinder (v1.8.0) with a doublet rate (dbr) of 0.1 and removed. Cell cycle scoring was performed using CellCycleScoring, and effects associated with the cell cycle, the number of counts, and the number of features were regressed out using the ScaleData function. Further analysis included PCA and UMAP dimensional reduction (1–30 PCs). Clustering was performed with FindNeighbors() and a resolution parameter of 0.7.

Mouse Adult Pancreatic Islet Data Integration with Seurat. Pre-processed datasets for Seurat integration contained 6,915; 564; 6,983; 22,800; 1,300; and 3,490 cells for the Chung, De Jesus, Tritschler, Kleiber, Baron and Wang et al. datasets, respectively. Data integration was performed using FindIntegrationAnchors() and IntegrateData() (1, 9), with 1–30 PCA dimensions. The integrated object was scaled using ScaleData(), and PCA was performed with RunPCA(). UMAP dimensional reduction (RunUMAP() with dims = 1:30) and clustering (FindClusters() with resolution = 1) were conducted, resulting in an integrated dataset of 42,052 cells and 19,961 features (SCTransform assay).

For subclustering analysis (**Supplementary Figure 1E**), we focused on cells expressing Stellate markers to identify progenitor-related populations. Cell IDs belonging to these populations were identified using Seurat’s WhichCells() function and retrieved from the original preprocessed objects. The subset normalized data (SCTransform) was re-integrated using Seurat. Data integration was performed using FindIntegrationAnchors() and IntegrateData() (1, 9), with 1–15 PCA dimensions. The integrated object was scaled using ScaleData(), and PCA was performed with RunPCA(). UMAP dimensional reduction (RunUMAP() with dims = 1:15) and clustering (FindClusters() with resolution = 0.2) were conducted, resulting in an integrated dataset of 580 cells and 19,955 features (SCTransform assay).

Mouse Adult Pancreatic Islet Data Integration with Conos. As an alternative integration method, Conos (v1.5.2) was used (**Figure 1**). Pre-processed datasets for Conos integration contained 6,779; 558; 6,850; 22,301; 1,298; and 3,263 cells for the Chung, De Jesus, Tritschler, Kleiber, Baron and Wang et al. datasets, respectively. After dimensional reduction in Seurat, 3,000 highly variable genes were selected. The Conos buildGraph() function was used with parameters k=15, k.self=5, space='PCA', ncomps=30, n.odgenes=2,000, matching.method = ‘mNN’, and metric = ‘angular’. Clustering was performed using the leiden.community method with resolution = 0.6 and 100 iterations. Graph embedding (embedGraph()) was conducted using the ‘largeVis’ method, resulting in an integrated dataset of 41,049 cells and 32,541 features (RNA assay).

For subclustering analysis (**Figure 1E**), we focused on cells expressing Stellate markers to identify progenitor-related populations. Cell IDs belonging to these populations were identified using Seurat’s WhichCells() function and retrieved from the original preprocessed objects. The subset normalized data (NormalizeData() with normalization.method=LogNormalize) was re-integrated using Conos. The Conos buildGraph() function was used with parameters k=30, k.self=5, space='PCA', ncomps=10, n.odgenes=2,000, matching.method = ‘mNN’, and metric = ‘angular’. Clustering was performed using the leiden.community method with resolution = 0.2 and 100 iterations. Graph embedding (embedGraph()) was conducted using the ‘largeVis’ method, resulting in an integrated dataset of 648 cells and 32,541 features (RNA assay).

Human Adult Pancreatic Islet Data Preprocessing and Integration. Human adult pancreatic islet data was obtained from the panc8 dataset (1). The following datasets were processed: Grün et al. (GSE81076) (10), Muraro MJ et al. (GSE85241) (11), Segerstolpe et al. (E-MTAB-5061) (12). Finally, the Wang et al. dataset, which contains the original mouse pancreatic islet cells already clustered, including the Procr^+^ progenitor population, was kindly provided by the authors as described above (8). SoupX was not applied to this analysis for RNA removal since the human datasets were sequenced using the CEL-Seq, CEL-Seq2 and SMARTSeq2 protocols, and SoupX is optimized for 10X Genomics dataset processing.

Unless otherwise specified, all samples were equally processed as follows: Cells with unique feature counts between the 10^th^ and 90^th^ percentiles for each sample, and mitochondrial percentages below 10% were retained. SCTransform was applied for normalization. Potential doublets were identified using scDbIFinder (v1.8.0) with a doublet rate (dbr) of 0.1 and removed. Cell cycle scoring was performed using CellCycleScoring, and effects associated with the cell cycle, the number of counts, and the number of features were regressed out using the ScaleData function. Further analysis included PCA and UMAP dimensional reduction (1–30 PCs). Clustering was performed with FindNeighbors() and a resolution parameter of 0.7.

Pre-processed datasets for Seurat integration contained 705; 1,046; 1,450; and 3,490 cells for the Grün, Muraro, Segerstolpe, and Wang et al. datasets, respectively. For integration of human and mouse pancreatic islet data (**Supplementary Figure 1O-T**), gene homologs between human and mouse were mapped. Data integration was performed using FindIntegrationAnchors() and IntegrateData() (1, 9), with 1–30 PCA dimensions. The integrated object was scaled using ScaleData(), and PCA was performed with RunPCA(). UMAP dimensional reduction (RunUMAP() with dims = 1:30) and clustering (FindClusters() with resolution = 0.5) were conducted, resulting in an integrated dataset of 7,291 cells and 21,329 features (SCTransform assay).

For subclustering analysis (**Supplementary Figure 1O**), we focused on cells expressing Stellate markers to identify progenitor-related populations. Cell IDs belonging to these populations were identified using Seurat’s WhichCells() function and retrieved from the original preprocessed objects. The subset normalized data (SCTransform) was re-integrated using Seurat. Data integration was performed using FindIntegrationAnchors() and IntegrateData() (1, 9), with 1–15 PCA dimensions. The integrated object was scaled using ScaleData(), and PCA was performed with RunPCA(). UMAP dimensional reduction (RunUMAP() with dims = 1:15) and clustering (FindClusters() with resolution = 0.2) were conducted, resulting in an integrated dataset of 427 cells and 21,203 features (SCTransform assay).

Mouse Embryonic Pancreas Data Integration with Conos. Single-cell RNA-seq 10X Cell Ranger output files, aligned to the mm10 mouse genome version, for the mouse embryonic pancreas dissected at different developmental stages, were downloaded from GEO with accession numbers GSM3140915 (E12.5), GSM3140916 (E14.5), and GSM3140917 and GSM3140918 (E17.5) (13); and GSM3938451 (E13.5) (14).

Before integration with Conos, cells were preprocessed using the Seurat package in R. Raw count matrices were loaded to create Seurat objects with a minimum threshold of 2–3 cells per feature and 200 features per cell. Quality control metrics included filtering cells with fewer than 1,000 or more than 5,000 features and excluding cells with mitochondrial gene percentages exceeding 5% or below 1%. For E13.5, cells with fewer than 500 features were excluded.

All datasets were normalized using the LogNormalize method with a scale factor of 10,000. Highly variable features (n = 3,000) were identified using the VST method. Dimensionality reduction was performed via principal component analysis (PCA), retaining the top 30 components for downstream analysis. The E17 datasets (GSM3140917 and GSM3140918) were integrated using the Seurat integration workflow. Integration anchors were identified using the FindIntegrationAnchors() function, followed by integration with the IntegrateData() function. The integrated dataset was scaled, PCA was repeated, and clustering was performed with a resolution of 0.5. Data visualization was conducted using UMAP.

Doublet detection and removal were performed for each individual dataset using the scDblFinder package (15), with a doublet rate (dbr) of 0.1. Cells identified as doublets were excluded, and the filtered singlet datasets were saved for downstream analysis. This process resulted in 11,373 cells for E12.5, 3,590 cells for E13.5, 7,882 cells for E14.5, and 10,188 cells for E17.5 samples of the mouse embryonic pancreas.

To account for cell cycle effects, cell cycle scoring was performed on the filtered singlet datasets using the CellCycleScoring() function in Seurat. Mouse homologs of the canonical S and G2/M phase genes were used. Cells were scored for S and G2/M phase gene expression, and the data were scaled using ScaleData(), regressing out cell cycle scores (S.Score and G2M.Score), UMI counts (nCount_RNA), and the number of features (nFeature_RNA).

Following preprocessing, datasets from the embryonic mouse pancreas and the Wang et al. adult pancreatic islet dataset (8) were integrated. Preprocessed datasets were integrated using the Conos tool (2). A shared nearest neighbor (SNN) graph was constructed in PCA space using 40 components and 2,000 over-dispersed genes per dataset. Parameters for graph building included a neighborhood size (k) of 30, a self-neighborhood size (k_self) of 10, and angular distance metrics. Clusters were identified using the Leiden algorithm with a resolution of 1, and dimensionality reduction was performed using largeVis for visualization.

To analyze in more detail the transcriptomes of pancreatic epithelial cells (clusters #6, 3, 19, 7, 4, 8, 12, 17, 18, and 5; **Supplementary Figure 2A, B**) and mesothelial cells with a Procr-like transcriptional profile (clusters #2 and 10; **Supplementary Figure 2A, B**), subsets of the integrated object were created in Seurat based on key pancreatic and progenitor markers (e.g., *Pdx1*, *Sox9*, *Neurog3*, *Procr*, *Rspo1*, *Lgals7*, *Upk3b*, *Clnd10*, and *Fgf1*). Selected cells were extracted from the original preprocessed datasets to generate new subset objects for E12.5, E13.5, E14.5, E17.5, and Procr^+^ progenitor cells from the adult pancreas dataset (Wang et al.) (8).

The integration of these subsets was performed using Conos. A shared nearest neighbor (SNN) graph was constructed in PCA space using 40 components and 2,000 highly variable genes per dataset. Integration parameters included a neighborhood size (k) of 30, a self-neighborhood size (k_self) of 10, self-neighborhood weight of 0.1; angular distance metrics, and Mutual nearest neighbors (mNN) matching method. To identify cellular communities, the Leiden algorithm was applied with a resolution of 1 and 100 iterations to optimize community detection. The final integrated object consisting of 23,190 cells was converted into a Seurat object for downstream analysis, which allowed the visualization and further assessment of cluster composition, gene expression, and biological relevance (**Figure 2A-C**).

The Slingshot trajectory inference package (16) was used to identify potential lineage relationships among cell populations within the integrated dataset (**Figure 2E**). Dimensionality reduction was performed using the largeVis algorithm, and cell clusters were assigned based on predefined cell type annotations. Lineage paths were identified with the getLineages() function, specifying "Mesothelial" as the starting cluster and "Beta" as the terminal cluster, reflecting the biological progression of interest. Lineages were visualized by overlaying them on the reduced dimensional space, facilitating the exploration of differentiation dynamics between progenitor-like populations and mature beta cells.

Mesothelial cells with a Procr-like transcriptional profile from each developmental stage dataset and the adult pancreas dataset were analyzed in detail (**Figure 2F-H**). Cells were identified using Seurat’s WhichCells() function, and integration was performed using Conos. The resulting graph used 40 components and 2,000 HVGs per dataset, with parameters including a neighborhood size (k) of 30, a self-neighborhood size (k_self) of 5, and angular distance metrics. The Leiden algorithm was applied to detect communities with a resolution of 0.5 over 100 iterations. Dimensionality reduction was performed using largeVis, and the integrated object was converted into a Seurat object for downstream analysis. Two minor clusters co-expressing pancreatic markers (e.g., *Pdx1*, *Sox9*, and/or *Ptf1a*) alongside erythrocyte markers (e.g., *Hba-a1*, *Hba-a2*) or endothelial markers (*Pecam1*) at high levels were discarded, as they were considered potential doublets that persisted after initial filtering. This integration strategy allowed for a comprehensive evaluation of mesothelial cell populations with a Procr-like transcriptional profile across development and adulthood.

Organoid scRNA-seq Analysis. The original mouse organoid dataset, containing pre-clustered cells including the Procr^+^ progenitor cell population and organoid stages 1 to 6, was kindly provided by the authors (8) and used as is, without additional processing other than the merging of specific organoid stages as described in the main text (**Figure 4A-C**). This dataset contained 20,901 features across 9,595 samples collected from the adult pancreatic islet dataset, as well as from Day 7 and Day 28 organoid stages, as outlined in the original report (8).

We retained the originally reported cell clustering, which consisted of Procr, Org.1, Org.2, Org.3, Org.4, Org.5, Org.6, β, α, δ, and PP cells. Clusters α, δ, and PP were excluded to focus the analysis on clusters connecting Procr progenitors to β-cells. Additionally, outlier cells from the Org.3, Org.4, and β-cell clusters (81 out of 7,196, 1.12%) were removed, as they were misclassified based on re-analysis of cluster markers. Organoid stages 1 and 2 (Org.1-2) and stages 3 to 5 (Org.3-5) were merged for downstream analysis due to their closely related global transcriptional profiles.

Data was further processed for visualization using Seurat (v4.4.0). Heatmaps were produced using the pheatmap R package, with the data scale adjusted to display expression levels based on lower and upper quantile thresholds (0.02 and 0.98, respectively).

Human and Mouse Embryonic Pancreas Cell Data Integration. We used raw single-cell RNA-seq data profiled from the human embryonic pancreas of the following post-conception week (PCW) embryonic stages: 4 (2 replicates), 5 (2 replicates), 6 (2 replicates), 7 (2 replicates), 8 (5 replicates), 9 (1 replicate), 10 (1 replicate), 11 (2 replicates) (17). Access to restricted raw data was granted by the authors thought the Genome Sequence Archive (GSA) for Human (study accession HRA002757). Raw *fastq* reads were aligned to the GRCh38 human genome version 32 (Ensembl 98) using the CellRanger (v.7.1.0) count function with default parameters. The initial downstream processing was performed as described in the original report (17). Briefly, this included the removal of ambient RNA contamination from each count matrix using SoupX (18) and downstream analyses with the Seurat package v4.3.0 (1). We kept cells with UMI counts between 4,000 and 50,000, nfeatures between 500-8,000, and mitochondrial count percentages <15%. The Seurat objects from the PCW 4-6 (W4-W6) and PCW 7-11 (W7-W11) samples were initially separately analyzed to keep epithelial cells, as described (17). This comprised normalization by the total counts per cell and selection of the 3,000 (W4-W6) and 2,000 (W7-W11) most variable genes for downstream analysis. The cell cycle score was regressed out, and Principal component analysis (PCA) was performed, followed by UMAP and Louvain clustering applied with the top 50 PCs and a resolution of 1 (W4-6) or 0.3 (W7-11). Doublets were detected and removed using the scDblFinder package (15), with doublet scores and classifications used to select singlets for further analysis. UMAP and dot plot visualizations over singlets were essential to subset interesting clusters (**Supplementary Figure 5A-D**). The human embryonic pancreas dataset contained 81,590 cells in total. Of these, 31,920 cells corresponded to embryonic stages spanning weeks 4, 5, and 6; while 49,670 cells corresponded to embryonic stages from weeks 7 to 11. Following the removal of doublets using the scDblFinder package (15), the resulting singlet objects contained 28,368 and 44,734 cells, respectively.

To focus the analysis on the pancreatic epithelial cells and the mesothelial cells with a Procr-like transcriptional profile, we next separately subset each object to keep these clusters (**Figure 5A-C**). For this purpose, we selected clusters # 34, 18, 10, 32, 27, 23, 14, 24, 29 and 33 (**Supplementary Figure 5A, B**) from the W4-6 object; clusters # 12, 7, 10, 1, 5, 13, 17, 18 and 4 (**Supplementary Figure 5C, D**) from the W7-11 object; and cell IDs from the mouse epithelial and mesothelial object described above (**Figure 2A**) to create new objects containing the mouse embryonic (E12.5, E13.5, E14.5, E17.5) cells of interest. We also created a new object with the adult mouse Procr^+^ progenitors from Wang et al. (8).

Prior to data integration, we converted the gene names of the mouse embryonic pancreas samples (E12.5, E13.5, E14.5 and E17.5) (13, 14), and of the Procr^+^ progenitor cells of the adult pancreatic islet dataset from Wang et al. (8) to the human nomenclature. We used for this purpose the same homology table used for integration of human and mouse islet data described above, consisting of 17,629 gene homologs between human and mouse.

Next, all mouse embryonic objects were re processed as follows. Samples were normalized using the LogNormalize method with a scale factor of 10,000. Highly variable features (n = 3,000) were identified using the VST method. Dimensionality reduction was performed via principal component analysis (PCA), retaining the top 30 components for downstream analysis. The object containing the adult Procr^+^ progenitor cells was similarly preprocessed, but using 1,000 variable features.

Preprocessed datasets from the human and mouse embryonic pancreas, and the Procr^+^ progenitor cells from the adult islet dataset (8) were next integrated using the Conos tool (2). A shared nearest neighbor (SNN) graph was constructed in PCA space using 30 components and 3,000 over-dispersed genes per dataset. Parameters for graph building included a neighborhood size (k) of 30, a self-neighborhood size (k_self) of 10, and angular distance metrics. Clusters were identified using the Leiden algorithm at a resolution of 1, and dimensionality reduction was performed using largeVis for visualization. Some clusters were then manually annotated according to the co-expression of well-known pancreatic progenitor or differentiated cell markers, and some clusters co-expressing genes coding for endocrine hormones were additionally subclustered using the Seurat package (**Figure 5A, B**).

We used the Slingshot trajectory inference package (16) to identify potential lineage relationships among cell populations within the integrated dataset (**Figure 5F**). Dimensionality reduction was performed using the largeVis algorithm, and cell clusters were assigned based on predefined cell type annotations. The getLineages function was used to determine lineage paths, specifying "Mesothelial^TBX3+ early^ " as the starting cluster and "Beta" as the terminal cluster to reflect the biological progression of interest. Lineages were represented as a SlingshotDataSet, and lineage trajectories were visualized by overlaying them on the reduced dimensional space. This approach facilitated the exploration of differentiation dynamics between progenitor-like populations and mature beta cells.

Human *In Vitro* Pancreatic Progenitor Data Analysis. The single-cell RNA-seq dataset for the human *in vitro*-derived Day 13 pancreatic progenitors is publicly available from the GEO with accession number GSM5127847 (19). Filtering, alignment to the GRCh38 human genome version 28 (Ensembl 92) and unique molecular identifier (UMI)-collapsing were performed using the Cell Ranger (v2.01) pipeline with default mapping arguments (10X Genomics), as described previously (19). Downstream analyses were run using the Seurat package v5.1. (1). To remove low-quality cells, we filtered cells with a high fraction of counts from mitochondrial genes (8% or more) or below 1%, indicating stressed or dying cells, and cells expressing fewer than 2,000 genes and 5,000 counts. Additionally, genes with expression in fewer than 10 cells were excluded. To improve the quality of the analysis, we also excluded outlier cells with >7000 genes detected or with less than 1% fraction of counts from mitochondrial genes.

Sample data were log-normalized, cell-cycle regressed and scaled. Clusters were identified with the Louvain algorithm using FindNeighbors and FindClusters commands in Seurat. Wilcoxon rank-sum tests were performed to identify differentially expressed genes in each cluster using the FindMarkers function in Seurat. Visualization in 2D was performed using the dimensionality reduction algorithm Uniform Manifold Approximation and Projection (UMAP). Doublet Detection and Removal was performed using the scDblFinder package (15), with a doublet rate (dbr) of 0.1. Cells identified as doublets were excluded, and the filtered singlet datasets were saved for downstream analysis. Cell clusters were then manually annotated according to the co-expression of well-known pancreatic progenitor or Procr^+^ progenitor markers (**Figure 5G-H**).

We next integrated the *in vitro* Procr-like cell subset from this analysis, with mesothelial cluster cells (Mesothelial/MPC, Mesothelial^TBX3+ early^, Mesothelial, Mesothelial pr. and Mesothelial^TBX3+ late^) of the W4-11 human embryonic pancreas (**Figure 5J-M**). For this purpose we recovered cell IDs in each cluster of interest from the original preprocessed objects, prior to integration in the case of the embryonic samples. Cells belonging to these populations were identified using Seurat’s WhichCells function. The integration was performed using the Conos package. A shared nearest neighbor (SNN) graph was constructed in PCA space, leveraging 40 components and 2,000 HVG per dataset. Integration parameters included a neighborhood size (k) of 30, a self-neighborhood size (k_self) of 10, and angular distance metrics. The Leiden algorithm was applied to detect communities with a resolution of 0.5 over 100 iterations. Dimensionality reduction was next performed using largeVis and the integrated object was converted into a Seurat object for downstream analysis.

**Single-cell ATAC-seq Data Processing and Analysis**

Single-cell CellRanger ATAC output files (aligned to the mm10 mouse genome version) for the E17.5 mouse embryonic pancreas were downloaded from the GEO with accession numbers GSM7244762 and GSM7244763 (20). Single-nucleus ATAC-seq data from embryonic mouse pancreas (E17.5) was processed and analyzed using Seurat (v5.1.0) and Signac (v1.14.0) packages in R (21).

scATAC-seq sample preprocessing. Samples from each lane were similarly preprocessed. Chromatin accessibility data was read from .h5 and .csv files to generate a chromatin assay with mm10 genome annotations, and a Seurat object with cells containing at least 200 features and features detected in at least 10 cells was created for further analyses. Quality control (QC) metrics, including nucleosome signal, TSS enrichment, and fraction of reads in peaks, were computed, and low-quality cells were excluded based on thresholds for these metrics and blacklist ratios.

The filtered dataset underwent normalization using TF-IDF, dimensionality reduction with singular value decomposition (SVD), and clustering through nearest-neighbor graph construction. UMAP was performed to visualize clusters. Doublets were identified and removed using the scDblFinder package, and singlet cells were retained for downstream analyses.

Peak information was extracted to generate a BED file for genomic annotation. Gene annotations were incorporated using the EnsDb.Mmusculus.v79 database. Final processed data, including QC-filtered, analyzed, and singlet Seurat objects, were saved as .rds files for reproducibility. Visualization and depth correlation were conducted to ensure high-quality data representation

Data Integration and Preprocessing. To integrate and analyze scATAC-seq data from two lanes of a mouse embryonic pancreas experiment, peak calling was performed independently for each lane. The resulting peak sets from lane 1 (L1) and lane 2 (L2) were loaded and converted into genomic ranges. A unified set of peaks was created by merging the two peak sets using the reduce function, and peaks were filtered based on length to retain high-confidence regions (20–10,000 bp).

Quality Control**.** Metadata for each lane was loaded, and cells with fewer than 500 passed filters were excluded. Additional filtering was applied to retain only high-quality singlets identified through Seurat object QC. This was accomplished by cross-referencing metadata with cell IDs from prefiltered Seurat objects.

Fragment and Feature Matrix Creation. Filtered metadata was used to create fragment objects for each lane. Peak counts were quantified for each dataset using the FeatureMatrix function, resulting in matrices of peaks by cells for each lane. Chromatin assays were constructed, and Seurat objects were created for each lane, ensuring metadata alignment.

Data Merging and Dimensionality Reduction. Seurat objects from the two lanes were merged into a single object, with unique cell IDs appended to indicate lane of origin. The merged dataset underwent preprocessing, including term frequency-inverse document frequency (TF-IDF) normalization, top feature selection, and singular value decomposition (SVD) for dimensionality reduction. UMAP was performed on the resulting dimensions (2–50).

Clustering and Annotation**.** The merged dataset was clustered using the Louvain algorithm with a resolution of 0.3, following neighbor graph construction on reduced dimensions. Clustering results were visualized using UMAP, and coverage plots of genomic regions were generated to validate data integrity. Peak annotation was performed using Ensembl annotations (EnsDb.Mmusculus.v79), ensuring compatibility with the mm10 genome.

Data Preprocessing and scRNA-seq Annotation. Epithelial scRNA-seq data integrated using CONOS was imported and subset to retain cells from embryonic stage E17 (object E17RNA_Conos). Identities were reassigned to descriptive labels reflecting known cell types. Standard Seurat RNA workflows were performed, including normalization, identification of variable features, and scaling of expression data for downstream integration. A curated set of markers was selected for visualizations, including UMAP and DotPlot representations.

Peak Annotation and Gene Activity Quantification in scATAC-seq Data. To annotate chromatin accessibility data, genome annotations were obtained using EnsDb.Mmusculus.v79. The scATAC-seq object was updated with these annotations and the UCSC-style chromosome naming convention. Gene activities were computed using the Seurat GeneActivity function, extending upstream and downstream of gene bodies, followed by normalization and scaling of these activities.

Label Transfer via Integration with scRNA-seq Data**.** Anchors for integration were identified using the FindTransferAnchors function with canonical correlation analysis (CCA) as the reduction method. Cell type predictions from scRNA-seq (E17RNA_Conos) were transferred to the scATAC-seq object based on gene activity profiles. Predicted cell identities were added as metadata to the scATAC-seq object, and the consistency between predictions and ground-truth cluster identities was evaluated.

Visualization of Integrated Data. UMAP projections were generated for the scATAC-seq object, displaying both predicted annotations (from scRNA-seq) and ground-truth cluster identities. Marker-specific DotPlots were constructed to assess the chromatin accessibility of selected features in predicted clusters. A custom color palette was applied to enhance contrast across cell types in all visualizations.

Motif Discovery. *De novo* motif discovery was performed with HOMER (22) using a window of 500 bp centered at the Mesothelial^Upk3b-low^ chromatin accessibility region cluster markers recovered from the scATAC-seq analysis (**Figure 3B**). The top scoring motifs ranked by P-value (setting a significant threshold of P-value ˂ 0.05) are shown in **Figure 3G**. Matching DNA binding motifs were associated to the *de novo* recovered matrix when the HOMER score was 0.7 or higher.

**Statistical analysis**

Statistical significance was assessed using Wilcoxon rank-sum test with *P* < 0.05 considered significant. All statistical analysis and visualization were done with R and Bioconductor package.

**Supplementary Figure legends**

**Supplementary Figure 1. Procr^+^ progenitors are not found in other adult pancreatic islet samples.**

(A) UMAP plots of single-cell transcriptomes profiled from six different mouse adult pancreatic islet datasets, integrated with Seurat. The bar below the UMAP displays the proportion of each cell type relative to the total number of cells analyzed. UMAP plots in the bottom show cell distribution according to the sample of origin.

(B) Dot plot showing the expression of key markers for endocrine, ductal, acinar, stellate, immune, and endothelial cell types, used to define clusters.

(C) Proportion of cells from each dataset contributing to the cell clusters identified in (A).

(D) UMAP plot highlighting the Procr^+^ progenitor cells originally reported.

(E) UMAP plot of pancreatic stellate cells reclustered from (A) and reanalyzed with Seurat.

(F) UMAP plot showing cells colored by the sample of origin. The DeJesus dataset was excluded from this analysis as it contributed an extremely small number of cells.

(G) Violin plots showing the expression of key active (*Pdgfra*, *Col3a1*, *Col1a1*) and inactive (*Pdgfrb*) stellate markers, along with Procr^+^ progenitor cell markers (*Procr*, *Rspo1*, *Upk3b*, among others), for each stellate cell subcluster identified in (E).

(H) UMAP plot highlighting the Procr^+^ progenitor cells originally reported.

(I) Proportion of cells from each dataset contributing to each stellate cell subcluster.

(J) Violin plots showing the expression of key Procr^+^ progenitor cell markers (*Procr*, *Rspo1*, *Upk3b*, among others), for each stellate cell subcluster identified in (E), split by the sample of origin.

(K) UMAP plot of single-cell transcriptomes profiled from three different datasets of human adult pancreatic islets and the mouse adult pancreatic islet sample from Wang and colleagues (8). Data was integrated with Seurat.

(L) Dot plot showing the expression of key markers for endocrine, ductal, acinar, stellate, immune, and endothelial cell types, used to define clusters.

(M) Proportion of cells from each dataset contributing to the cell clusters identified in (K).

(N) UMAP plot highlighting the Procr^+^ progenitor cells originally reported.

(O) UMAP plot of pancreatic stellate cells reclustered from (K) and reanalyzed with Seurat.

(P) UMAP plot showing cells colored by the sample of origin. The Grun dataset was excluded from this analysis as it contributed an extremely small number of cells.

(Q) Violin plots showing the expression of key activated (*PDGFRA*, *COL3A1*, *COL1A1*) and quiescent (*PDGFRB*) stellate markers, along with Procr^+^ progenitor cell markers (*PROCR*, *RSPO1*, *UPK3B*, among others), for each stellate cell subcluster identified in (O).

(R) UMAP plot highlighting the Procr^+^ progenitor cells originally reported.

(S) Proportion of cells from each dataset contributing to each stellate cell subcluster.

(T) Violin plots showing the expression of key Procr^+^ progenitor cell markers (*PROCR*, *RSPO1*, *UPK3B*, among others), for each stellate cell subcluster identified in (O), split by the sample of origin.

**Supplementary Figure 2. Mesothelial cells in the mouse embryonic pancreas exhibit a transcriptional profile closely resembling that of adult Procr^+^ progenitors.**

(A) Dimension plot of single-cell transcriptomes profiled from the E12.5-E17.5 mouse embryonic pancreas, integrated with the originally reported mouse adult pancreatic islet dataset from Wang and colleagues (8). Data integrated using Conos.

(B) Dot plot showing the expression of selected pancreatic and non-pancreatic markers used to match cell clusters. Cells clustered as in (A).

(C) Dimension plot highlighting the location of the originally reported Procr^+^ progenitors.

(D) Dimension plots in the bottom panel show cell distribution according to sample of origin.

(E) Feature plots showing the expression of selected mesothelial and Procr^+^ progenitor cell markers.

(F) Dimension plot (dimensions 2 and 3; related to Fig. 2A) of single-cell pancreatic epithelial and mesothelial transcriptomes profiled from the E12.5-E17.5 mouse embryonic pancreas, integrated with the originally reported Procr^+^ progenitor cells profiled from adult pancreatic islets.

(G, H) Feature plots showing the expression of selected BP/ductal cell markers in clustered cells of the epithelial and mesothelial transcriptome analysis (G; refer for Fig. 2A for cell cluster labels in the original dimension plot) and in all cells clustered from the mouse embryonic pancreas (H; refer to panel A for cell cluster labels in the original dimension plot).

(I) tSNE plot of single-cell transcriptomes as originally labeled by Wang and colleagues (8) from the adult mouse pancreatic islets containing the Procr^+^ progenitor cells.

(J) Violin plots showing the expression of key pancreatic and Procr^+^ progenitor/mesothelial cell markers in cells as originally clustered by Wang and colleagues (I).

**Supplementary Figure 3. The chromatin accessibility landscape of a subset of mesothelial cells supports epithelial pancreatic progenitor competence.**

(A) UMAP plot of single-cell accessibility profiles obtained for the E17.5 mouse embryonic pancreas.

(B) Dot plot showing the gene activity scores of selected pancreatic and non-pancreatic markers.

(C) Chromatin accessibility landscape in the vicinity of *Upk3b*, *Neurog3* and *Pdx1* for E17.5 pancreatic epithelial and mesothelial cells clustered as in (Fig. 3B). The bottom tracks display the fragment count for selected clusters, and the accessibility regions with enrichment over background (peaks). The violin plots on the right display the gene activity score for these genes at each cell cluster. Red boxes highlight gene promoters, well-known *Pdx1* enhancer regions (Area IV and Areas I-III), as well as other potential regulatory regions for *Upk3b* and/or *Upk3bl1*.

(D) UMAP plot of single-cell accessibility profiles from the Mesothelial^Upk3b-low^ cells subset from Fig. 3B and reclustered.

(E) Dot plot showing the gene activity scores of selected pancreatic and non-pancreatic markers. Data clustered as in (D).

(F) Violin plots showing the expression, in epithelial and mesothelial cells of the E17.5 embryonic pancreas, of selected genes coding for transcription factors recognizing the *de novo* motifs enriched at the Mesothelial^Upk3b-low^ marker regions. Cell cluster labels as in Fig. 2A.

(G) Violin plots showing the expression, in all cells of the E17.5 embryonic pancreas, of selected genes coding for transcription factors recognizing the *de novo* motifs enriched at the Mesothelial^Upk3b-low^ marker regions. Cell cluster numbers as in Fig. S2A.

(H) Violin plots showing the expression, in adult mouse pancreatic islet cells as originally profiled and clustered by Wang and colleagues (8). Cell cluster labels as in Fig. S2I.

**Supplementary Figure 4. Islet-like organoids differentiated *in vitro* from adult Procr^+^ progenitors mimic the transcriptional profile of the mesothelial and ductal/BP clusters in mouse pancreas development.**

(A) Dot plot showing the gene activity scores, in epithelial and mesothelial cells of the E17.5 embryonic pancreas, of selected genes coding for transcription factors recognizing the *de novo* motifs enriched at the Mesothelial^Upk3b-low^ marker regions. Cell cluster labels as in Fig. 3B.

(B) Chromatin accessibility landscape in the vicinity of *Lurap1l*, *Cp* and *Errfi* (organoid transitional genes enriched in embryonic ductal/BP cells) for E17.5 pancreatic epithelial and mesothelial cells clustered as in (Fig. 3B). The bottom tracks display the fragment count for selected clusters, and the accessibility regions with enrichment over background (peaks). The violin plots on the right display the gene activity score for these genes at each cell cluster.

**Supplementary Figure 5. *Procr-like* mesothelial cells are also identified in the human embryonic pancreas.**

(A, C) UMAP plot of single-cell transcriptomes profiled from the W4-W6 (A) or W7-11 (C) human embryonic pancreas, which were initially separately analyzed as explained in detail in the original report (17).

(B, D) Dot plot showing the expression of selected well-known pancreatic progenitor and non-epithelial cell type markers used to name cell clusters in the W4-W6 (B) or W7-11 (D) human embryonic pancreas. Color intensity indicates mean expression (normalized) in a cluster, dot size indicates the proportion of cells in a cluster expressing the gene. In panel B, pancreatic cells (dashed red ellipse in A) were defined as cells expressing high levels of either *PDX1*, *SOX9* or *PTF1A*. BP cells expressed *PDX1* and *SOX9*, were negative for *PTF1A* and presented high expression of the pro-endocrine markers *SOX4* or *RFX6*. Hepatoblasts, clearly clustered apart, presented the highest levels of well-known liver markers such as *AFP* and *APOA2*.

(E) Dimension plot (dimensions 2 and 3; related to Fig. 5A) of integrated single-cell pancreatic epithelial and mesothelial transcriptomes profiled from the E12.5-E17.5 mouse embryonic pancreas, the W4-W11 human embryonic pancreas, and Procr^+^ progenitors profiled from mouse adult islets.

(F) Violin plots showing the expression of *PROCR* for each cell cluster identified in Fig. 5A.

(G) Dimension plots (dimensions 1 and 2, and 2 and 3; related to Fig. 5A) showing just clustered cells of the W7-8 human embryonic pancreas.

**References**

1. Butler, A., Hoffman, P., Smibert, P., Papalexi, E., and Satija, R., Integrating single-cell transcriptomic data across different conditions, technologies, and species. *Nat Biotechnol* 36 (2018) **36**: 411-420. doi: 10.1038/nbt.4096

2. Barkas, N., Petukhov, V., Nikolaeva, D., Lozinsky, Y., Demharter, S., Khodosevich, K., and Kharchenko, P.V., Joint analysis of heterogeneous single-cell RNA-seq dataset collections. *Nature Methods* 16 (2019) **16**: 695-698. doi: 10.1038/s41592-019-0466-z

3. Chung, K.M., Singh, J., Lawres, L., Dorans, K.J., Garcia, C., Burkhardt, D.B., Robbins, R., Bhutkar, A., Cardone, R., Zhao, X., Babic, A., Vayrynen, S.A., Dias Costa, A., Nowak, J.A., Chang, D.T., Dunne, R.F., Hezel, A.F., Koong, A.C., Wilhelm, J.J., Bellin, M.D., Nylander, V., Gloyn, A.L., McCarthy, M.I., Kibbey, R.G., Krishnaswamy, S., Wolpin, B.M., Jacks, T., Fuchs, C.S., and Muzumdar, M.D., Endocrine-Exocrine Signaling Drives Obesity-Associated Pancreatic Ductal Adenocarcinoma. *Cell* 181 (2020) **181**: 832-847.e18. doi: 10.1016/j.cell.2020.03.062

4. de Jesus, D.S., Mak, T.C.S., Wang, Y.-F., von Ohlen, Y., Bai, Y., Kane, E., Chabosseau, P., Chahrour, C.M., Distaso, W., Salem, V., Tomas, A., Stoffel, M., Rutter, G.A., and Latreille, M., Dysregulation of the Pdx1/Ovol2/Zeb2 axis in dedifferentiated β-cells triggers the induction of genes associated with epithelial-mesenchymal transition in diabetes. *Molecular Metabolism* 53 (2021) **53**: 101248. doi: 10.1016/j.molmet.2021.101248

5. Tritschler, S., Thomas, M., Böttcher, A., Ludwig, B., Schmid, J., Schubert, U., Kemter, E., Wolf, E., Lickert, H., and Theis, F.J., A transcriptional cross species map of pancreatic islet cells. *Mol Metab* 66 (2022) **66**: 101595. doi: 10.1016/j.molmet.2022.101595

6. Kleiber, T., Davidson, G., Mengus, G., Martianov, I., and Davidson, I., Single cell transcriptomics reveal trans-differentiation of pancreatic beta cells following inactivation of the TFIID subunit Taf4. *Cell Death & Disease* 12 (2021) **12**: 790. doi: 10.1038/s41419-021-04067-y

7. Baron, M., Veres, A., Wolock, S.L., Faust, A.L., Gaujoux, R., Vetere, A., Ryu, J.H., Wagner, B.K., Shen-Orr, S.S., Klein, A.M., Melton, D.A., and Yanai, I., A Single-Cell Transcriptomic Map of the Human and Mouse Pancreas Reveals Inter- and Intra-cell Population Structure. *Cell Syst* 3 (2016) **3**: 346-360 e4. doi: 10.1016/j.cels.2016.08.011

8. Wang, D., Wang, J., Bai, L., Pan, H., Feng, H., Clevers, H., and Zeng, Y.A., Long-Term Expansion of Pancreatic Islet Organoids from Resident Procr(+) Progenitors. *Cell* 180 (2020) **180**: 1198-1211 e19. doi: 10.1016/j.cell.2020.02.048

9. Stuart, T., Butler, A., Hoffman, P., Hafemeister, C., Papalexi, E., Mauck, W.M., Hao, Y., Stoeckius, M., Smibert, P., and Satija, R., Comprehensive Integration of Single-Cell Data. *Cell* 177 (2019) **177**: 1888-1902.e21. doi: https://doi.org/10.1016/j.cell.2019.05.031

10. Grün, D., Muraro, M.J., Boisset, J.-C., Wiebrands, K., Lyubimova, A., Dharmadhikari, G., van den Born, M., van Es, J., Jansen, E., Clevers, H., de Koning, Eelco J.P., and van Oudenaarden, A., De Novo Prediction of Stem Cell Identity using Single-Cell Transcriptome Data. *Cell Stem Cell* 19 (2016) **19**: 266-277. doi: 10.1016/j.stem.2016.05.010

11. Muraro, M.J., Dharmadhikari, G., Grun, D., Groen, N., Dielen, T., Jansen, E., van Gurp, L., Engelse, M.A., Carlotti, F., de Koning, E.J., and van Oudenaarden, A., A Single-Cell Transcriptome Atlas of the Human Pancreas. *Cell Syst* 3 (2016) **3**: 385-394 e3. doi: 10.1016/j.cels.2016.09.002

12. Segerstolpe, A., Palasantza, A., Eliasson, P., Andersson, E.M., Andreasson, A.C., Sun, X., Picelli, S., Sabirsh, A., Clausen, M., Bjursell, M.K., Smith, D.M., Kasper, M., Ammala, C., and Sandberg, R., Single-Cell Transcriptome Profiling of Human Pancreatic Islets in Health and Type 2 Diabetes. *Cell Metab* 24 (2016) **24**: 593-607. doi: 10.1016/j.cmet.2016.08.020

13. Byrnes, L.E., Wong, D.M., Subramaniam, M., Meyer, N.P., Gilchrist, C.L., Knox, S.M., Tward, A.D., Ye, C.J., and Sneddon, J.B., Lineage dynamics of murine pancreatic development at single-cell resolution. *Nat Commun* 9 (2018) **9**: 3922. doi: 10.1038/s41467-018-06176-3

14. Campbell, S.A., McDonald, C.L., Krentz, N.A.J., Lynn, F.C., and Hoffman, B.G., TrxG Complex Catalytic and Non-catalytic Activity Play Distinct Roles in Pancreas Progenitor Specification and Differentiation. *Cell Reports* 28 (2019) **28**: 1830-1844.e6. doi: 10.1016/j.celrep.2019.07.035

15. Germain, P.L., Lun, A., Garcia Meixide, C., Macnair, W., and Robinson, M.D., Doublet identification in single-cell sequencing data using scDblFinder. *F1000Res* 10 (2021) **10**: 979. doi: 10.12688/f1000research.73600.2

16. Street, K., Risso, D., Fletcher, R.B., Das, D., Ngai, J., Yosef, N., Purdom, E., and Dudoit, S., Slingshot: cell lineage and pseudotime inference for single-cell transcriptomics. *BMC Genomics* 19 (2018) **19**: 477. doi: 10.1186/s12864-018-4772-0

17. Ma, Z., Zhang, X., Zhong, W., Yi, H., Chen, X., Zhao, Y., Ma, Y., Song, E., and Xu, T., Deciphering early human pancreas development at the single-cell level. *Nature Communications* 14 (2023) **14**: 5354. doi: 10.1038/s41467-023-40893-8

18. Young, M.D., and Behjati, S., SoupX removes ambient RNA contamination from droplet-based single-cell RNA sequencing data. *Gigascience* 9 (2020) **9**. doi: 10.1093/gigascience/giaa151

19. El-Khairi, R., Olszanowski, E., Muraro, D., Madrigal, P., Tilgner, K., Chhatriwala, M., Vyas, S., Chia, C.Y., Vallier, L., and Rodríguez-Seguí, S.A., Modelling HNF1B-associated monogenic diabetes using human iPSCs reveals an early stage impairment of the pancreatic developmental program. *Stem Cell Reports* 16 (2021) **16**: 2289-2304. doi:

20. de la O, S., Yao, X., Chang, S., Liu, Z., and Sneddon, J.B., Single-cell chromatin accessibility of developing murine pancreas identifies cell state-specific gene regulatory programs. *Mol Metab* 73 (2023) **73**: 101735. doi: 10.1016/j.molmet.2023.101735

21. Stuart, T., Srivastava, A., Madad, S., Lareau, C.A., and Satija, R., Single-cell chromatin state analysis with Signac. *Nature Methods* 18 (2021) **18**: 1333-1341. doi: 10.1038/s41592-021-01282-5

22. Heinz, S., Benner, C., Spann, N., Bertolino, E., Lin, Y.C., Laslo, P., Cheng, J.X., Murre, C., Singh, H., and Glass, C.K., Simple Combinations of Lineage-Determining Transcription Factors Prime cis-Regulatory Elements Required for Macrophage and B Cell Identities. *Molecular Cell* 38 (2010) **38**: 576-589. doi: 10.1016/j.molcel.2010.05.004
