# Supplementary figures and images for: Identification of mouse and human embryonic pancreatic cells with adult Procr+ progenitor transcriptomic and epigenomic characteristics
Source: Front Endocrinol (Lausanne). 2025 Feb 13;16:1543960. doi: 10.3389/fendo.2025.1543960 (PMC11864936; doi:10.3389/fendo.2025.1543960)

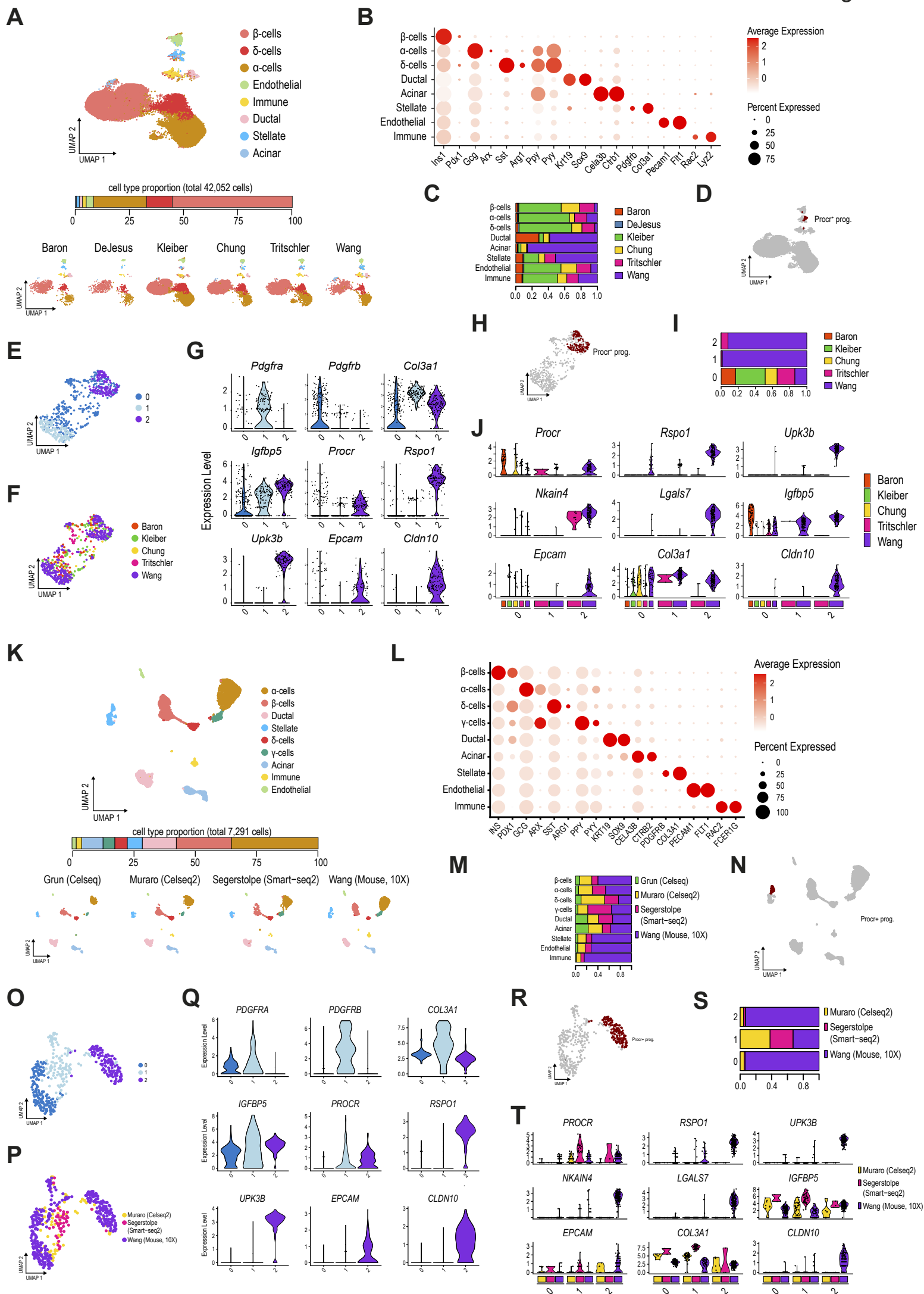

Supplement: Supplementary file 3 [file Image1.pdf]

**A**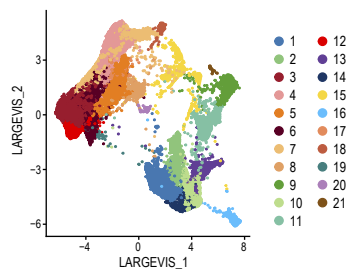**B**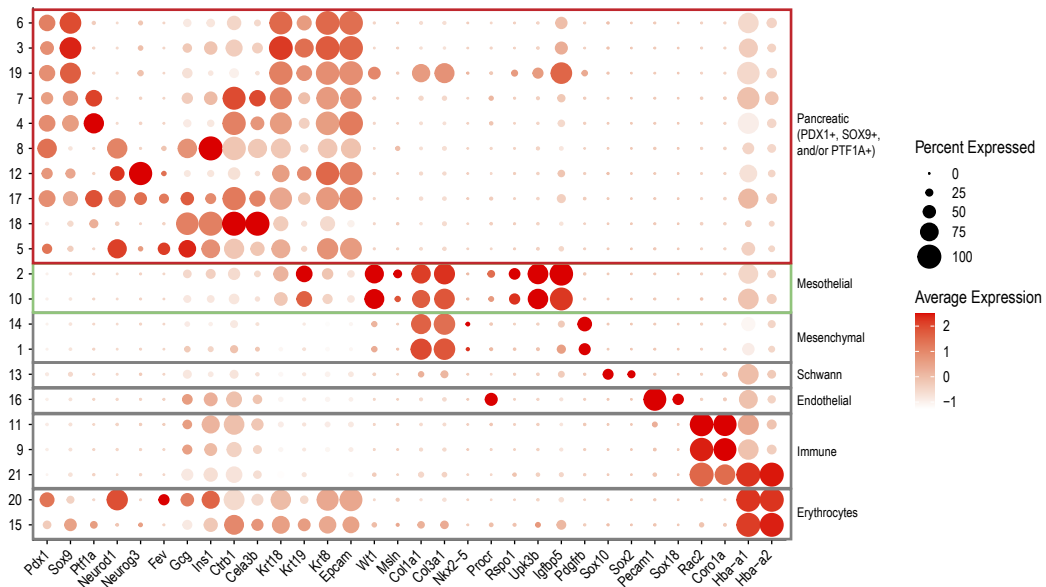**C**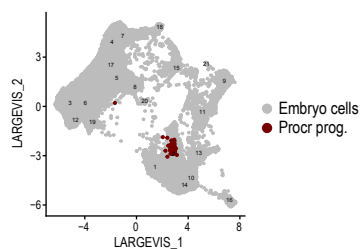**D**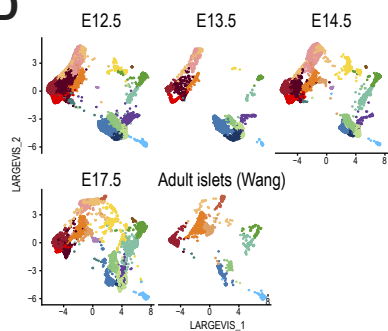**F**

E12.5-E17.5 epi. + Mesothelial + Procr prog.

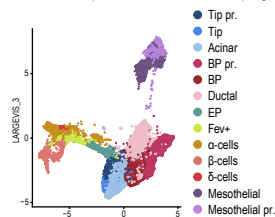**G**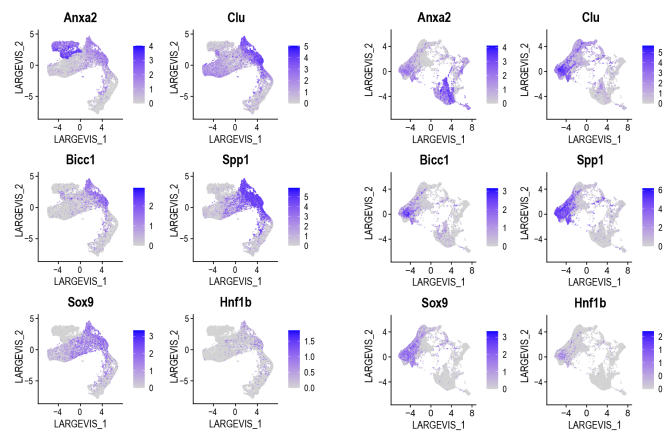**H****E**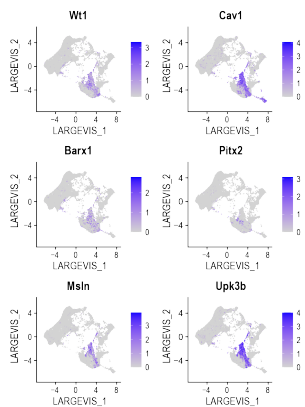**I**

Adult islets (Wang et al. data)

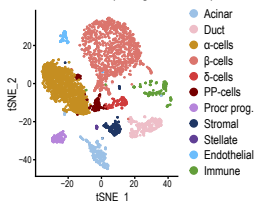**J**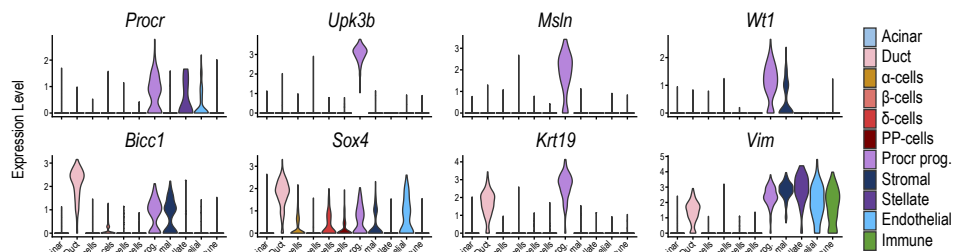

Supplement: Supplementary file 4 [file Image2.pdf]

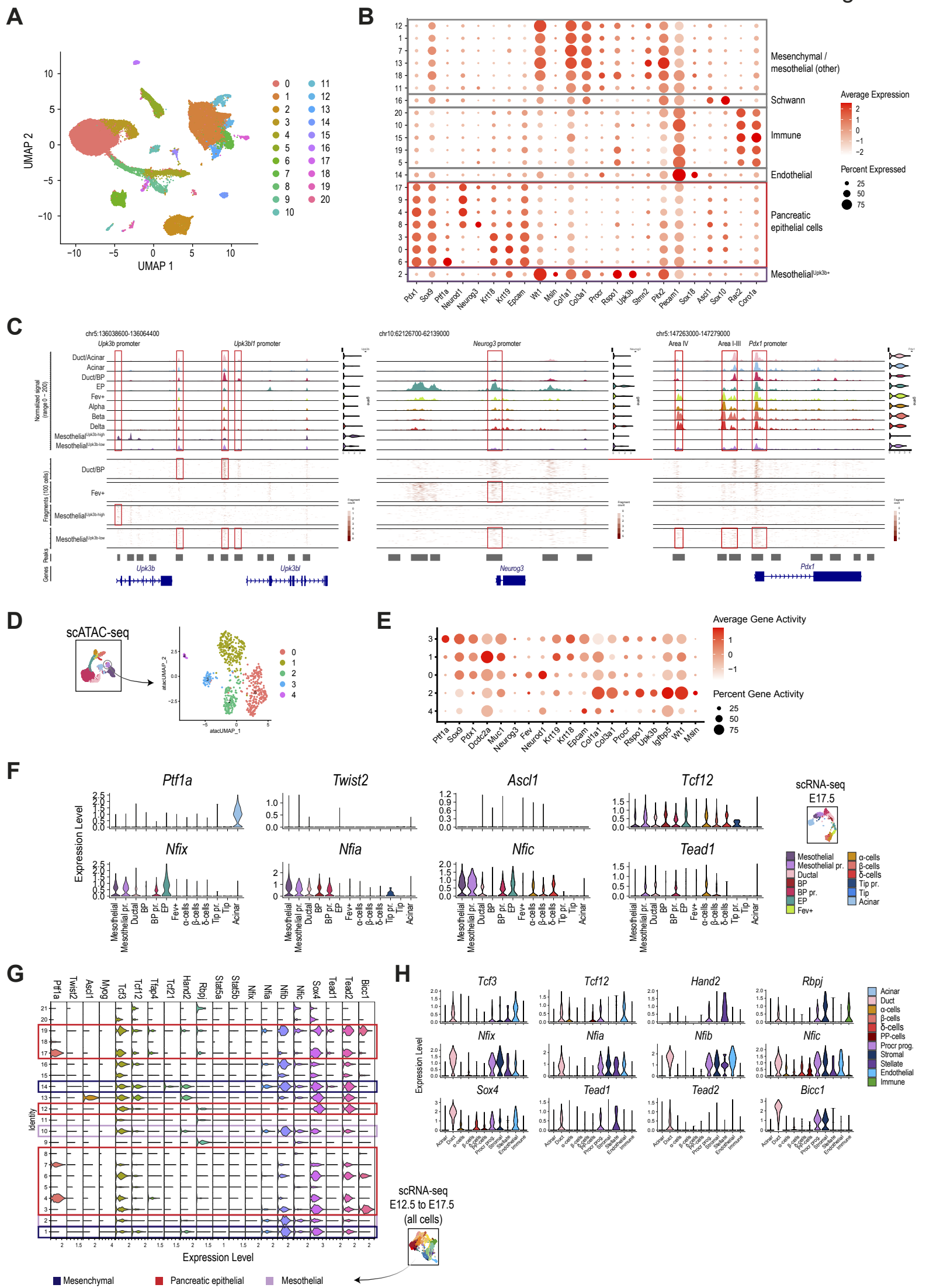

Supplement: Supplementary file 5 [file Image3.pdf]

A

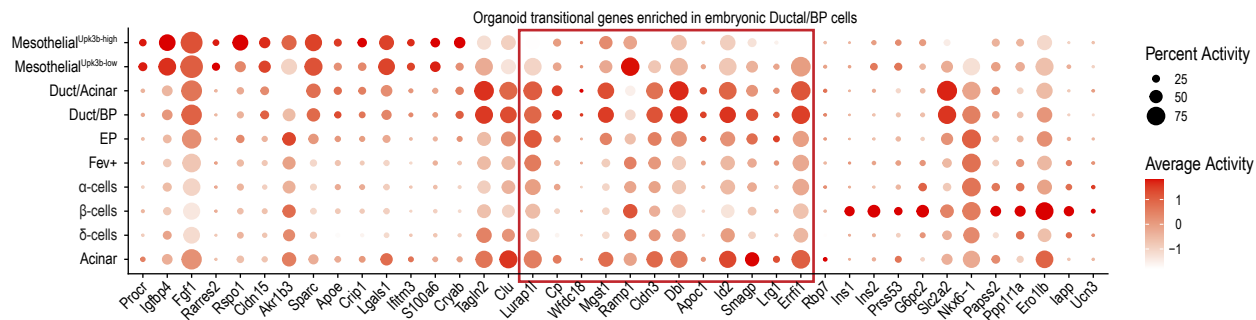

B

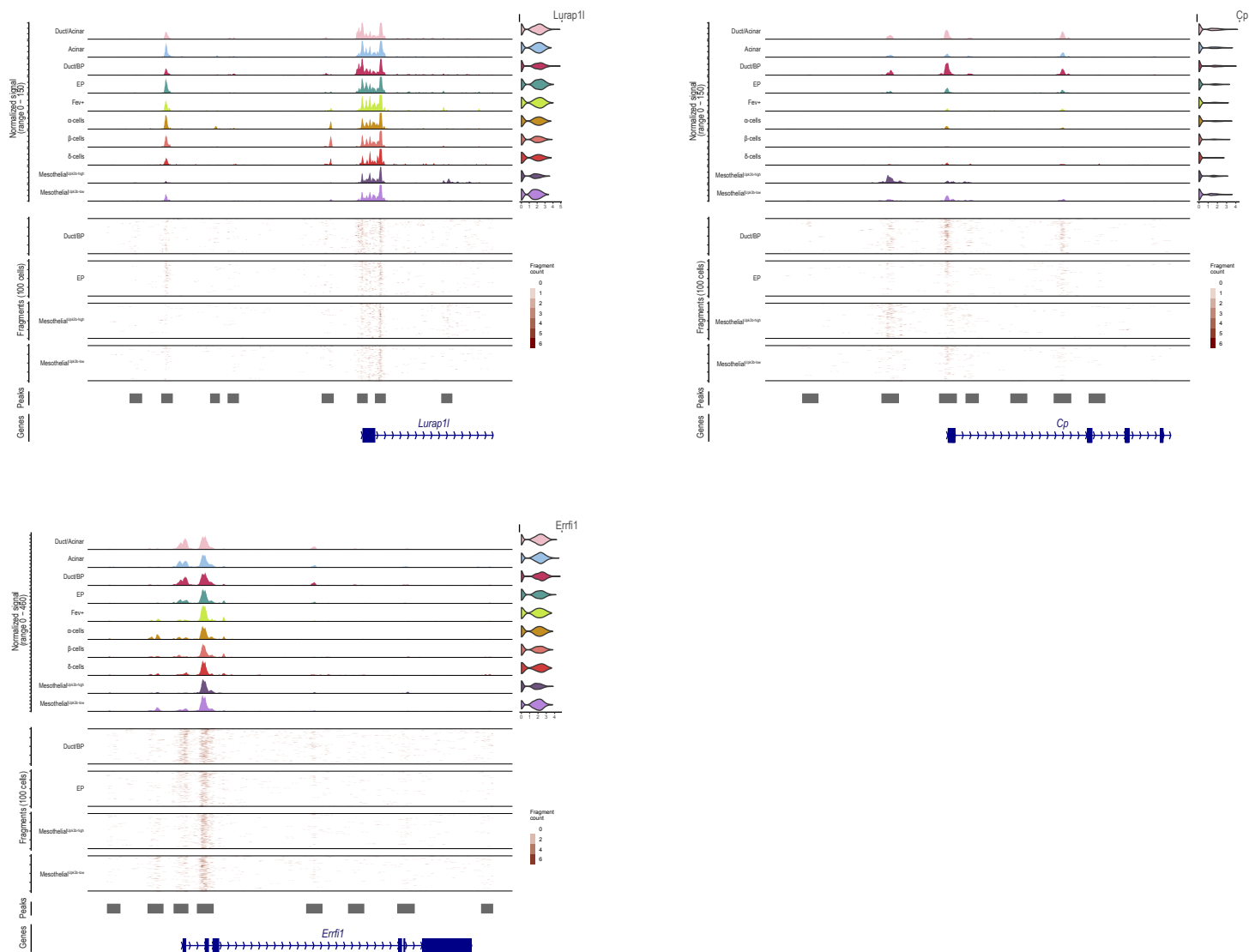

Supplement: Supplementary file 6 [file Image4.pdf]

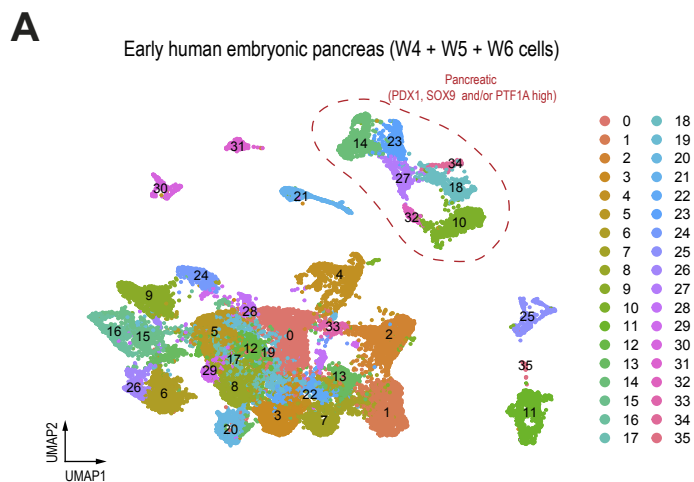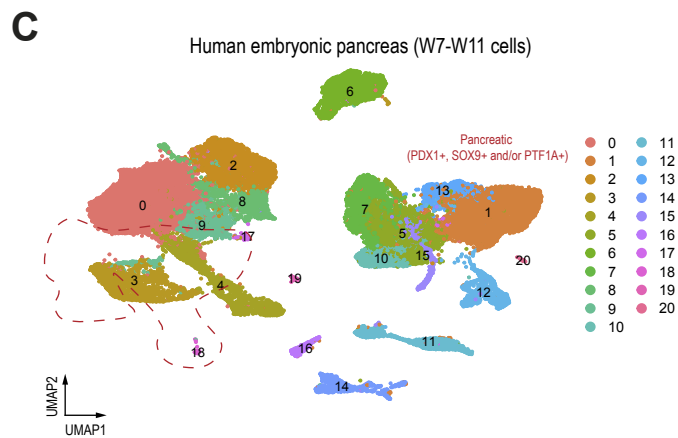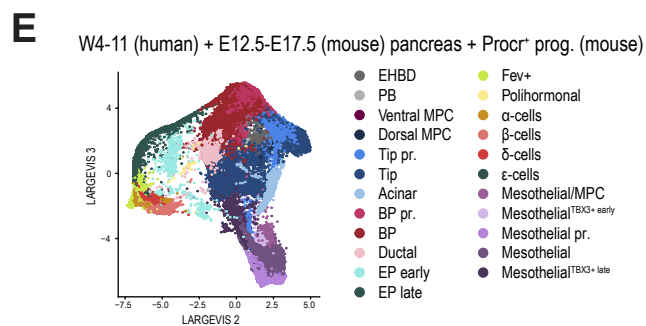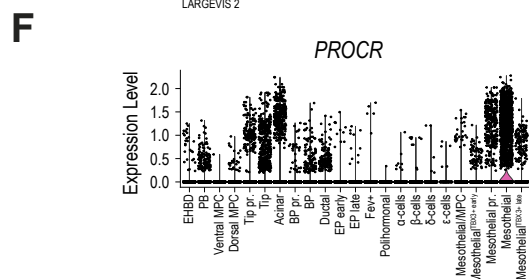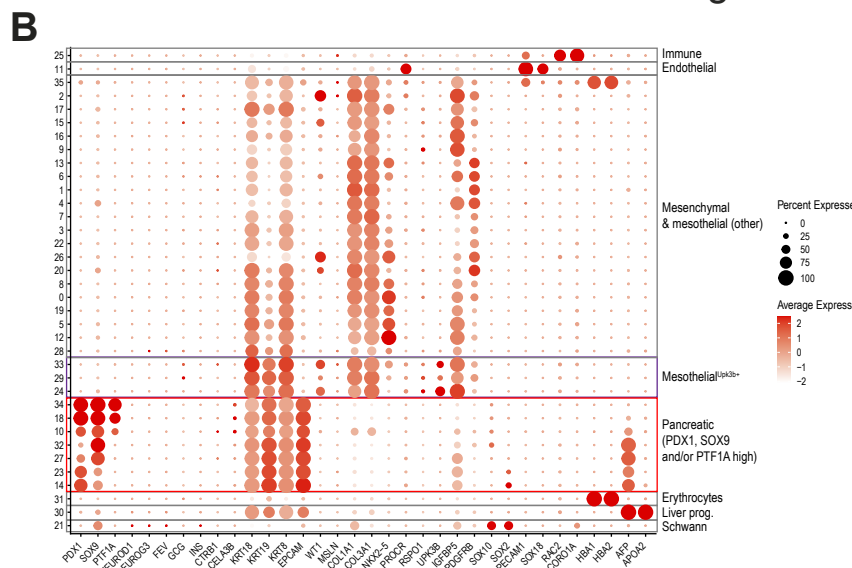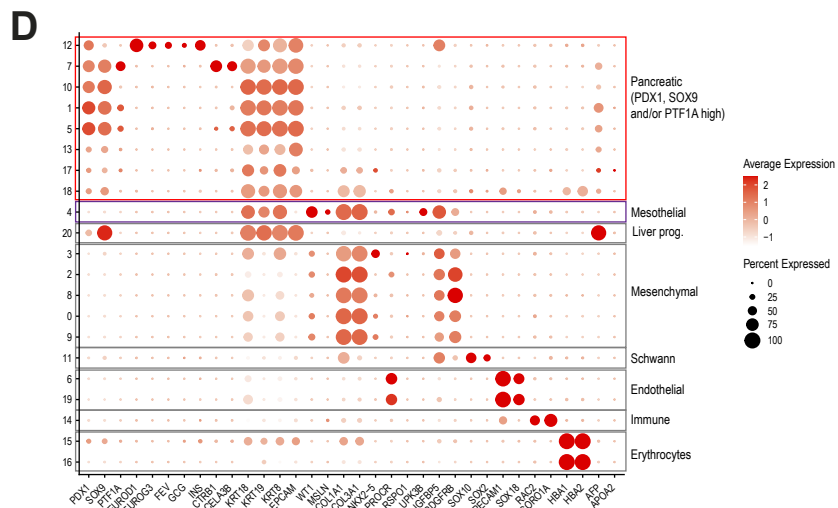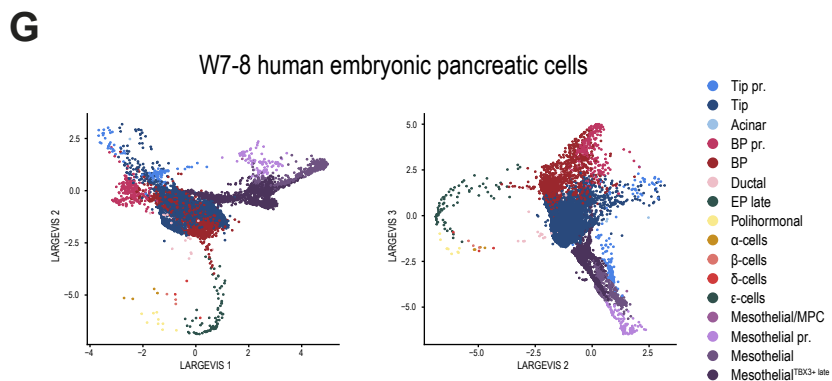

Supplement: Supplementary file 7 [file Image5.pdf]
